# Probiotics supplements for the prevention of atopic dermatitis in children: an umbrella review

**DOI:** 10.3389/fnut.2025.1587348

**Published:** 2025-07-15

**Authors:** Ling Zhong, Jia Su, Xiyuan Zhou, Huiying Wan

**Affiliations:** ^1^Department of Dermatology, Sichuan Provincial People’s Hospital, School of Medicine, University of Electronic Science and Technology of China, Chengdu, China; ^2^School of Medicine and Life Science, Chengdu University of Traditional Chinese Medicine, Chengdu, China

**Keywords:** probiotics, atopic dermatitis, children, umbrella, meta-analysis, prevention

## Abstract

**Introduction:**

Previous meta-analyses of multiple studies have suggested that probiotics supplementation plays a role in reducing the risk of atopic dermatitis (AD). However, the conclusions of these studies remain controversial.

**Methods:**

We conducted an umbrella review of meta-analyses to comprehensively analyze and evaluate the evidence regarding the association between probiotics and AD. We searched PubMed, Web of Science, Embase, Spous, and Cochrane Library databases for meta-analyses and systematic reviews up to October 2024. Our selection criteria encompassed meta-analyses of cohort studies, case–control studies, and randomized controlled clinical trials investigating the associations between probiotics and the risk of AD. We also assessed the levels of evidence for these associations using the AMSTAR 2 criteria.

**Results:**

A total of 32 eligible articles, including 126 meta-analyses, were included for qualitative synthesis in this umbrella review. The results indicate that probiotics supplementation is associated with a reduced risk of AD. The subgroup analysis indicates that supplementation with *Lactobacillus* spp., single-strain, and multi-strain probiotics is associated with a reduced risk of AD, with multi-strain formulations potentially demonstrating more pronounced effects. Furthermore, both combined prenatal and postnatal supplementation, as well as postnatal supplementation alone, contribute to a reduction in AD risk.

**Discussion:**

Probiotics supplementation may help reduce the risk of AD, with early-life administration playing a key role. Future research should focus on well-designed randomized controlled trials that account for potential sources of bias in order to provide evidence-based public health recommendations.

**Systematic review registration:**

PROSPERO (International00 Prospective Register of Systematic Reviews) under the registration number CRD42024599789. The publicly accessible registration record is available at: https://www.crd.york.ac.uk/PROSPERO/view/CRD42024599789.

## 1 Introduction

Atopic dermatitis (AD) is a chronic, recurrent inflammatory skin disease affecting 10–20% of children worldwide (1), considered the onset of the atopic process. Some children may develop asthma and allergic rhinitis, affecting their growth, development, and overall health in infancy and early childhood (2–4). Additionally, it increases the economic burden on their families. The rapid increase in AD prevalence globally, especially in developed countries, underscores the urgent need for primary prevention strategies (5, 6). As AD typically begins in infancy, this period may represent a critical window for intervention. The developing immune system and gut microbiota in children may be particularly responsive to probiotics modulation, potentially enhancing preventive effects.

*Lactobacillus* spp. and *Bifidobacterium* spp. may modulate immune function via toll-like receptors (TLRs), potentially contributing to mucosal homeostasis and the prevention of AD (7, 8). Therefore, the World Health Organization suggests that administering live probiotics in appropriate doses and at optimal timing may contribute to the prevention of allergic diseases (9). The exact mechanisms by which probiotics prevent AD remain unclear. Increasing research has explored early-life probiotics supplementation as a preventive strategy for atopic diseases, but findings remain inconsistent. The optimal strains, timing, and potential adverse effects are yet to be fully determined. The American Academy of Pediatrics maintains a cautious stance on using probiotics for preventing atopic diseases, stressing the need for further evidence before recommending routine use (10). Therefore, a systematic and comprehensive approach is necessary to gain a clearer understanding of the relationship between probiotics and the risk of AD.

Umbrella reviews have been widely utilized to systematically analyze and assess meta-analyses, particularly in examining the relationships between various factors (such as nutrition, risk factors, and behaviors) and health outcomes. This approach enhances the reliability and precision of findings (11–14). To better understand and reassess this association, we conducted an umbrella review of all available meta-analyses. This study may serve as a foundation for future research in broader populations, including adults, pregnant women, and the elderly.

## 2 Materials and methods

The protocol and registration details for this umbrella review have been pre-registered with PROSPERO (International Prospective Register of Systematic Reviews) under the registration number CRD42024599789. The publicly accessible registration record is available at: https://www.crd.york.ac.uk/PROSPERO/view/CRD42024599789. This study adheres to the Preferred Reporting Items for Systematic Reviews and Meta-Analyses (PRISMA) reporting guidelines (15).

### 2.1 Literature search strategy

We conducted a systematic search of PubMed, Web of Science, Embase, Spous, and Cochrane Library databases for systematic reviews and meta-analyses published from database inception to October 2024 on the association between probiotics supplementation and the risk of AD. The search strategy included the following keyword combinations: “(probiotics OR probiotics OR prebiotics OR prebiotic OR synbiotics OR synbiotic OR postbiotic OR postbiotics OR microbiological supplements) AND (“dermatitis, atopic” OR “atopic dermatitis” OR “eczema, atopic” OR “atopic eczema” OR “neurodermatitis, atopic” OR “atopic neurodermatitis” OR “neurodermatitis, disseminated” OR “disseminated neurodermatitis” OR “eczema, infantile” OR “infantile eczema”) AND (“systematic review” OR “systematic literature review” OR “meta-analysis” OR “meta analysis”).”There were no language restrictions. Relevant studies were identified and screened based on titles, abstracts, and full texts. To reduce the risk of language-related publication bias, non-English articles were included if they met the eligibility criteria and had sufficient methodological clarity. When necessary, professional translation tools (e.g., DeepL, ChatGPT) or assistance were used to extract data from these studies.

### 2.2 Eligibility and inclusion/exclusion criteria

The included studies were meta-analyses assessing the association between probiotics supplementation and the risk of AD. The specific inclusion criteria were as follows: (i) Meta-analyses of cohort studies, case–control studies, or randomized controlled trials (RCTs) investigating the effect of probiotics supplementation on the risk of AD. (ii) Considering the incidence of AD as the study outcome. (iii) Reporting effect sizes (OR, odds ratio; RR, relative risk; HR, hazard ratio; RD, risk difference) and corresponding confidence intervals (CIs). (iv) Oral probiotics are formulations that contain one or more strains of beneficial bacteria. (v) The control group received a placebo. The exclusion criteria were as follows: (i) Studies without original data to calculate the pooled risk estimates and 95% CIs. (ii) Systematic reviews without a meta-analysis. (iii) Articles, letters, editorials, and conference abstracts. (iv) Duplicate publications.

### 2.3 Data extraction and quality assessment

Data extraction was conducted independently by two investigators, followed by verification by a third researcher. In cases of disagreement, a fourth investigator made the final decision. From each eligible meta-analysis, we extracted the following information: first author, year of publication, type of probiotics, timing of probiotics supplementation, number of included studies, study design of the original research, number of cases and participants, adjusted effect estimates, corresponding 95% confidence intervals (CIs), and heterogeneity results (I^2^). For the original studies included in the systematic reviews or meta-analyses, we extracted the first author, number of cases and participants, effect estimates, and corresponding 95% CIs for further analysis.

We assessed the methodological quality of each meta-analysis using the Assessment of Multiple Systematic Reviews, version 2 (AMSTAR-2) tool. This tool has been proven to be a reliable and effective method for evaluating the quality of systematic reviews and meta-analyses (16). We used Egger’s regression test to assess publication bias and excluded studies with significant bias. Then, we applied the Trim and Fill method to adjust the effect size and conducted a sensitivity analysis by comparing the results before and after adjustment (17).

### 2.4 Statistical analysis

For each individual meta-analysis, we re-applied both the fixed-effects model and the random-effects model to calculate the pooled effect size and the corresponding 95% confidence intervals (CIs) (18). The I^2^ statistic was used to assess heterogeneity across studies (19). Additionally, we calculated the 95% confidence interval for I^2^ to evaluate the uncertainty in heterogeneity assessment (20). Furthermore, we computed the 95% prediction intervals (PIs) for the pooled effect size under the random-effects model. This metric provides additional insights into between-study heterogeneity and indicates the uncertainty of the expected effect size in future studies examining the same association (21). The 95% PIs represent the range within which the true effect sizes of 95% of similar studies are expected to fall in potential future pooled analyses or studies conducted in comparable populations (22). We used Egger’s test to assess publication bias of each meta-analysis (23). A *p*-value < 0.05 in Egger’s test indicated the presence of small-study effects, meaning that the estimate from the largest component study (i.e., the study with the smallest standard error) was more conservative than the summary estimate from the random-effects model (24–28).

We assessed excess significance bias by determining whether the observed number of studies (O) with nominally statistically significant results (*p* < 0.05) exceeded the expected number (E) (29). For each meta-analysis, E was estimated as the sum of the statistical power of all component studies. To approximate the power of individual studies, we typically used the effect size from the largest study within the meta-analysis (29, 30). We applied a noncentral t-distribution to assess the statistical power of each study (29). An excess significance bias was considered present if the *p*-value was <0.10, indicating that O exceeded E.

Moreover, subgroup evaluation was carried out based on the type of probiotics supplementation (e.g., *Lactobacillus* spp., *Bifidobacterium* spp., prebiotics, synbiotics, single-strain, mixed-strains) and the timing of probiotics supplementation (e.g., prenatal, postnatal, prenatal and postnatal). Prenatal probiotics intervention involves maternal oral supplementation of probiotics during pregnancy (typically in the second or third trimester) until delivery, aiming to modulate the fetal immune system indirectly. Postnatal probiotics intervention refers to the administration of probiotics directly to the infant after birth and/or continued maternal supplementation, which may influence the infant via breast milk. Combined prenatal and postnatal intervention entails maternal supplementation beginning during pregnancy and continuing postpartum through the mother and/or infant, targeting immunomodulation during both fetal development and early infancy. Finally, we evaluated the incidence of adverse events associated with probiotics. To assess potential heterogeneity arising from study design, we conducted subgroup analyses stratified by study type [randomized controlled trials (RCTs) vs. cohort studies]. This allowed us to evaluate whether the observed associations varied meaningfully between different research designs.

### 2.5 Assessment of evidence credibility

The assessment of evidence strength was based on the following criteria (17, 22, 27, 31–34): (i) *p* < 10^−6^ in a random-effects meta-analysis; (ii) a sample size exceeding 1,000 participants; (iii) *p* < 0.05 in the largest individual study; (iv) between-study heterogeneity with I^2^ < 50%; (v) no indication of small-study effects; (vi) a 95% prediction interval that excluded the null value; and (vii) no evidence of excess significance bias. Using these criteria, associations were categorized into five levels of evidence: convincing (Class I), highly suggestive (Class II), suggestive (Class III), weak (Class IV), and non-significant. Evidence was classified as convincing if all seven criteria were met. If (i)–(iii) criteria were satisfied, the classification was highly suggestive. When only the criteria of *p* ≤ 0.001 under a random-effects model and a sample size >1,000 were met, the evidence was considered suggestive. If only the criterion of *p* ≤ 0.05 under a random-effects model was met, the classification was weak. Evidence was deemed not significant when the *p*-value exceeded 0.05 under a random-effects model. All statistical analyses were conducted using Stata (version 15.0) and R studio (version 4.3.2). Apart from the predefined cutoff values, statistical significance was set at *p* < 0.05 (two-tailed).

### 2.6 Overlap assessment and strategy for handling overlapping meta-analyses

To assess the degree of overlap among the included meta-analyses, we calculated the Corrected Covered Area (CCA) using the following formula (35):


$$\mathrm{CCA}=\frac{Nr-\mathrm{Ns}}{(R*\mathrm{Ns})-\mathrm{Ns}}$$

where Nr is the total number of primary study occurrences (including duplicates), Ns is the number of unique primary studies, and R is the number of meta-analyses. The CCA quantifies the proportion of overlap beyond what would be expected by chance, and the degree of overlap is interpreted as follows: 0–5% (slight), 6–10% (moderate), 11–15% (high), and >15% (very high) (35). This metric helps identify redundancy across meta-analyses and potential bias due to duplicated evidence.

Based on the Corrected Covered Area (CCA) assessment, we adopted different strategies to address the overlap among included meta-analyses. When the degree of overlap was high (CCA ≥ 6%), two approaches were considered: (1) selecting only one or a few representative meta-analyses for further analysis, prioritizing the most recent, most relevant, or most comprehensive in terms of included primary studies (36, 37); or using established quality assessment tools (e.g., AMSTAR-2) to identify and retain only the highest-quality reviews (38, 39). (2) extracting and merging all relevant primary studies from the existing meta-analyses to conduct a *de novo* analysis (40). When overlap was low (CCA ≤ 5%), the risk of bias from duplicated data was considered minimal, and the pooled estimates from existing meta-analyses were used directly for further analysis (40).

## 3 Results

**Figure 1:**
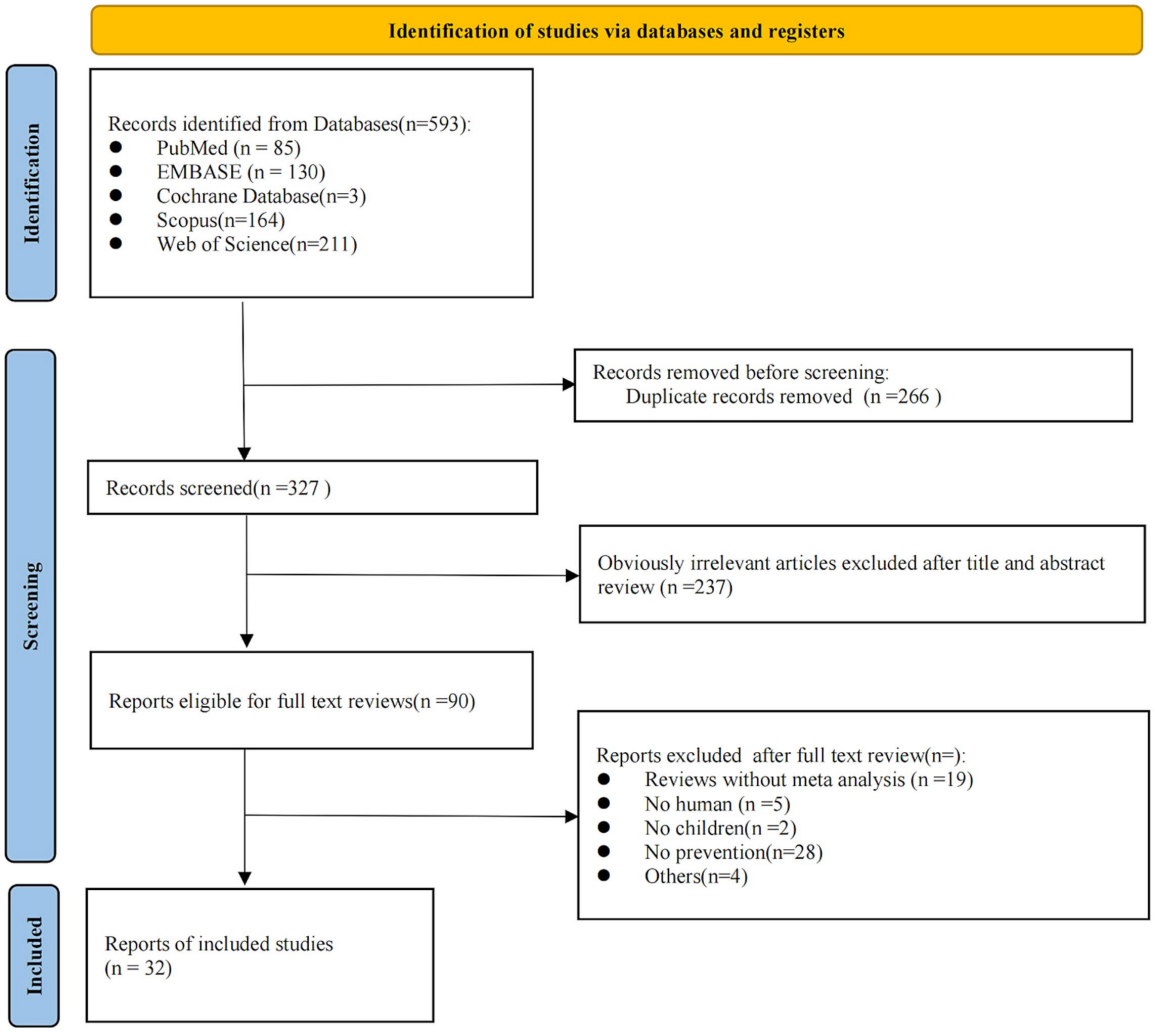
Flow chart of the literature search.

**Figure 2:**
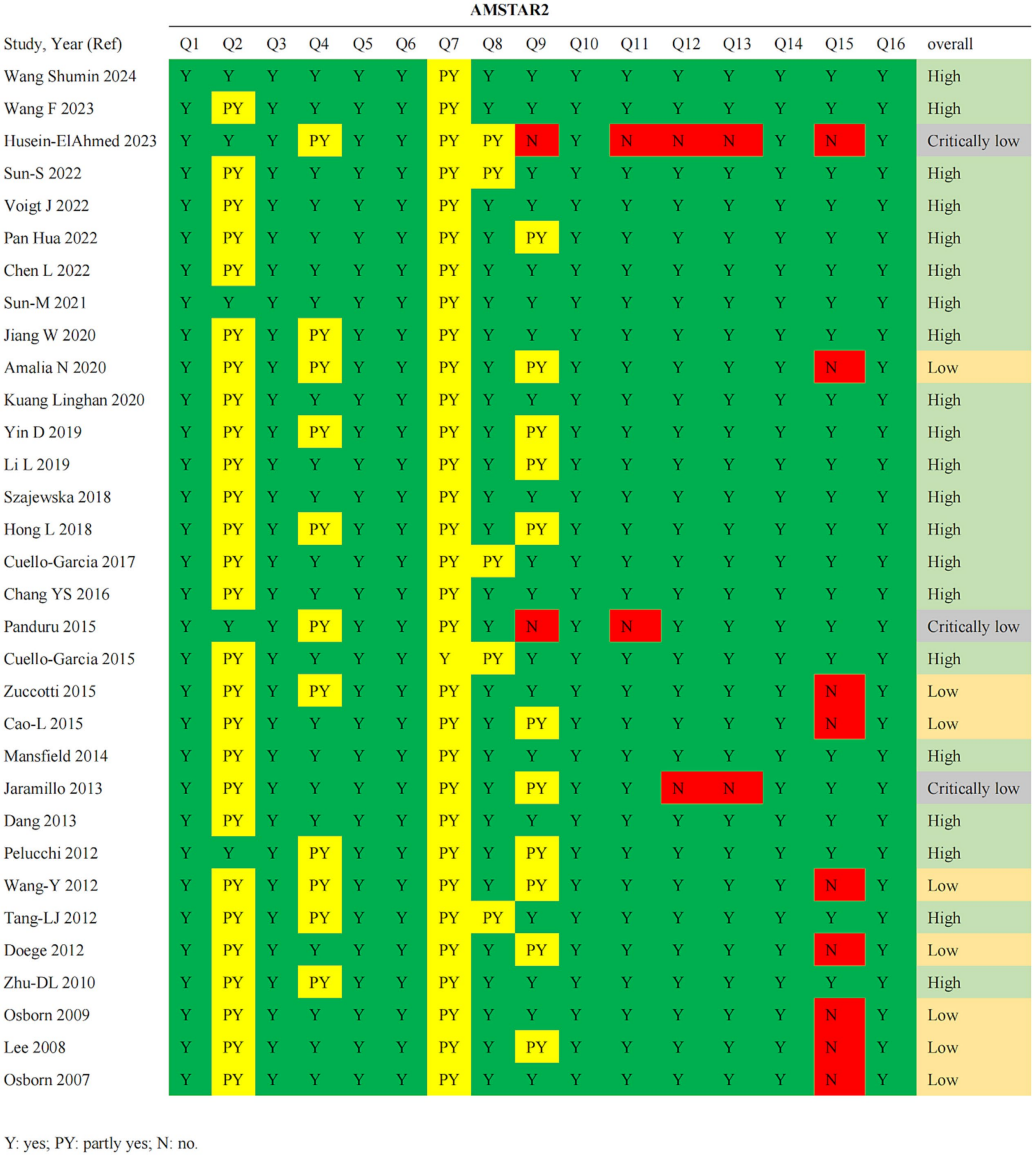
Results of risk of bias assessment based on AMSTAR 2 tool.

A total of 593 records were identified. After removing duplicates and screening titles and abstracts, 503 articles were excluded, and 90 references were selected for full-text evaluation. Ultimately, 32 studies comprising 126 comparisons were included in this umbrella review (Figure 1). Seven of the included articles were non-English [one in Spanish (41) and six in Chinese (42–47)]. AI-assisted tools, including ChatGPT and DeepL, were used to aid in comprehension and data extraction from these studies. In terms of the quality of the included meta-analyses, results from the AMSTAR 2 questionnaire showed that the present umbrella meta-analysis included 21 studies assessed as high quality, 8 studies as low quality, and 3 studies as critically low quality (Figure 2). A total of 126 comparisons of the included meta-analyses were reported in all eligible meta-analyses, with 119 examining the relationship between probiotics supplementation and AD outcomes (Table 1), and 7 investigating the association between probiotics supplementation and adverse events (Table 2). Subgroup analyses were also conducted based on the type of probiotics (e.g., *Lactobacillus* spp., *Bifidobacterium* spp., prebiotics, synbiotics, single-strain, mixed-strains) and the timing of supplementation (e.g., prenatal, postnatal, prenatal and postnatal). Finally, the incidence of adverse reactions associated with probiotics was evaluated. Notably, all studies in Table 2 are also included in Table 1.

**Table 1:** Summary of the meta-analyses of probiotics and AD risk.

Study year (ref)	Number of study	Study design	Exposure	Time	Cases/total	Type of metrics	Summary effect size (95% CI)	I^2^
Wang Shumin 2024 (102)	13 (7)	RCT	Probiotics		820/3147	OR	0.72 (0.62, 0.85)	17%
Wang F 2023 a (46)	29	RCT	Probiotics		1718/6154	RR	0.83 (0.73, 0.94)	65.2%
Wang F 2023 b (46)	18	RCT	Probiotics	Prenatal and postpartum	1364/4401	RR	0.64 (0.49, 0.85)	66.6%
Wang F 2023 c (46)	11	RCT	Probiotics	Postpartum	396/1977	RR	0.85 (0.62, 1.17)	44.7%
Wang F 2023 d (46)	8	RCT	*Lactobacillus rhamnosus*		517/2255	RR	0.54 (0.36, 0.80)	68.2%
Wang F 2023 e (46)	8	RCT	*Lactobacillus* spp.		295/1118	RR	1.09 (0.79, 1.49)	25.7%
Wang F 2023 f (46)	13	RCT	Mixed probiotics		447/3143	RR	0.70 (0.52, 0.93)	52.4%
Husein-ElAhmed 2023 a (103)	17	RCT	Probiotics	Postpartum	560/2844	OR	0.78 (0.64, 0.94)	53%
Husein-ElAhmed 2023 b (103)	7	RCT	Probiotics	Prenatal and postpartum	347/1298	OR	0.51 (0.39, 0.66)	64%
Husein-ElAhmed 2023 c (103)	14	RCT	Probiotics	Prenatal and postpartum	892/3602	OR	0.73 (0.63, 0.86)	43%
Husein-ElAhmed 2023 d (103)	5	RCT	Probiotics	Prenatal and postpartum	172/951	OR	0.71 (0.43, 0.86)	0%
Husein-ElAhmed 2023 e (103)	23	RCT	Single-strain		1010/3963	RR	0.80 (0.69, 0.93)	–
Husein-ElAhmed 2023 f (103)	20	RCT	Mixed-strains		1019/4876	RR	0.60 (0.52, 0.70)	–
Sun-S 2022 a (104)	17	RCT	Probiotics		1361/4011	OR	0.59 (0.45, 0.78)	69%
Sun-S 2022 b (104)	9	RCT	Single-strain		430/1761	OR	0.75 (0.55, 1.03)	45%
Sun-S 2022 c (104)	3	RCT	Single-strain		96/431	OR	0.97 (0.62, 1.54)	0%
Sun-S 2022 d (104)	8	RCT	Mixed-strains		931/2250	OR	0.44 (0.28, 0.71)	81%
Sun-S 2022 e (104)	3	RCT	Mixed-strains		126/762	OR	0.48 (0.32, 0.72)	0%
Sun-S 2022 f (104)	7	RCT	Probiotics	Prenatal and postpartum	840/2076	OR	0.74 (0.57, 0.97)	37%
Sun-S 2022 g (104)	3	RCT	Probiotics	Prenatal and postpartum	88/567	OR	0.73 (0.40, 1.35)	37%
Sun-S 2022 h (104)	5	RCT	Probiotics	Postpartum	159/908	OR	0.73 (0.41, 1.30)	56%
Voigt J 2022 (60)	10	RCT	*Lactobacillus rhamnosus*		620/2572	RR	0.6 (0.47, 0.75)	48%
Pan Hua 2022 (105)	8	RCT	Probiotics		968/2575	RR	0.86 (0.78, 0.95)	32.2%
Chen L 2022 a (106)	22	RCT	Probiotics		1395/5019	RR	0.74 (0.64, 0.86)	55.4%
Chen L 2022 b (106)	7	RCT	Probiotics	Postpartum	225/1181	RR	0.76 (0.60, 0.96)	–
Chen L 2022 c (106)	14	RCT	Probiotics	Prenatal and postpartum	1092/3628	RR	0.73 (0.61, 0.88)	–
Chen L 2022 d (106)	10	RCT	*Lactobacillus* spp.		491/1913	RR	0.84 (0.72, 0.98)	–
Chen L 2022 e (106)	11	RCT	Single-strain		823/2789	RR	0.64 (0.51, 0.80)	–
Sun-M 2021 a (65)	10	RCT	Probiotics		598/2093	RR	0.60 (0.47, 0.78)	67%
Sun-M 2021 b (65)	8	RCT	Probiotics	Prenatal	522/1588	RR	0.59 (0.45, 0.78)	71%
Sun-M 2021 c (65)	2	RCT	Probiotics	Postpartum	76/505	RR	0.63 (0.26, 1.48)	63%
Jiang W 2020 a (64)	16	RCT	Probiotics		879/3111	RR	0.70 (0.57, 0.84)	65%
Jiang W 2020 b (64)	7	RCT	Single-strain		368/1408	RR	0.84 (0.66, 1.09)	50%
Jiang W 2020 c (64)	9	RCT	Mixed-strains		511/1703	RR	0.61 (0.47, 0.78)	65%
Jiang W 2020 d (64)	8	RCT	Probiotics	Prenatal and postpartum	336/1340	RR	0.71 (0.58, 0.86)	18%
Jiang W 2020 e (64)	4	RCT	Probiotics	Postpartum	257/823	RR	0.88 (0.59, 1.33)	74%
Amalia N 2020 a (48)	32	RCT, cohort	Probiotics		5551/33192	RR	0.77 (0.70, 0.86)	58.5%
Amalia N 2020 b (48)	10	RCT, cohort	Probiotics	Prenatal and postpartum	592/2032	RR	0.75 (0.65, 0.87)	4.1%
Amalia N 2020 c (48)	9	RCT, cohort	Probiotics	Prenatal and postpartum	4248/28471	RR	0.87 (0.76, 0.98)	46.9%
Amalia N 2020 d (48)	8	RCT	Probiotics	Prenatal and postpartum	442/1504	RR	0.72 (0.52, 1.00)	74.8%
Amalia N 2020 e (48)	4	RCT	Probiotics	Postpartum	180/943	RR	0.85 (0.58, 1.25)	43.8%
Amalia N 2020 f	4	RCT	*L. rhamnosus* HN001		306/1233	RR	0.75 (0.62, 0.92)	5.5%
Amalia N 2020 g (48)	3	RCT	Bifidbacterium animalis HN019		276/868	RR	0.82 (0.67, 1.01)	0%
Amalia N 2020 h (48)	5	RCT	*Lactobacillus rhamnosus* GG		215/735	RR	1.04 (0.83, 1.30)	0%
Amalia N 2020 i (48)	2	RCT	*L. paracasei* F19		46/291	RR	0.55 (0.32, 0.97)	0%
Amalia N 2020 j (48)	18	RCT, cohort	Mixed-strains		4714/30065	RR	0.72 (0.62, 0.83)	71.5%
Kuang Linghan 2020 a (78)	9	RCT	Probiotics		599/3256	RR	0.68 (0.58, 0.81)	0%
Kuang Linghan 2020 b (78)	5	RCT	Probiotics		403/1348	RR	0.79 (0.68, 0.91)	27.6%
Yin D 2019 a (42)	22	RCT	Probiotics		1848/6561	RR	0.81 (0.70, 0.93)	65%
Yin D 2019 b (42)	10	RCT	*Lactobacillus* spp.		568/1981	RR	0.78 (0.73, 1.04)	50%
Yin D 2019 c (42)	11	RCT	Mixed-strains		1255/4510	RR	0.68 (0.52, 0.90)	78%
Yin D 2019 d (42)	6	RCT	Probiotics	Postpartum	251/933	RR	0.99 (0.80, 1.23)	44%
Yin D 2019 e (42)	16	RCT	Probiotics	Prenatal and postpartum	1430/5116	RR	0.77 (0.66, 0.90)	67%
Li L 2019 a (59)	28	RCT, cohort	Probiotics		2174/6892	OR	0.69 (0.58, 0.82)	53.6%
Li L 2019 b (59)	8	RCT, cohort	Probiotics	Postpartum	349/1358	OR	0.77 (0.59, 1.01)	38%
Li L 2019 c (59)	19	RCT, cohort	Probiotics	Prenatal and postpartum	1747/5324	OR	0.67 (0.54, 0.82)	61%
Li L 2019 d (59)	6	RCT, cohort	*L. rhamnosus*		321/1048	OR	0.65 (0.50, 0.86)	19%
Li L 2019 e (59)	15	RCT, cohort	Mixed-strains		1578/4636	OR	0.64 (0.51, 0.81)	62%
Szajewska 2018 a (107)	3	RCT	*Lactobacillus rhamnosus* GG	Prenatal and postpartum	106/352	RR	0.93 (0.49, 1.76)	72%
Szajewska 2018 b (107)	2	RCT	*Lactobacillus rhamnosus* GG	Prenatal and postpartum	71/236	RR	0.74 (0.43, 1.26)	44%
Hong L 2018 a (47)	20	RCT	Probiotics		1176/3701	RR	0.74 (0.64, 0.86)	57%
Hong L 2018 b (47)	13	RCT	Probiotics	Prenatal and postpartum	801/2540	RR	0.68 (0.57, 0.82)	59%
Hong L 2018 c (47)	2	RCT	Probiotics	Prenatal	118/314	RR	0.91 (0.62, 1.33)	41%
Hong L 2018 d (47)	5	RCT	Probiotics	Postpartum	257/847	RR	0.91 (0.71, 1.16)	25%
Hong L 2018 e (47)	5	RCT	*Lactobacillus* spp.		263/986	RR	0.70 (0.52, 0.94)	38%
Hong L 2018 f (47)	7	RCT	Mixed-strains		509/1484	RR	0.65 (0.50, 0.86)	74%
Cuello-Garcia 2017 (79)	6	RCT	Prebiotics		341/2030	RR	0.68 (0.40, 1.15)	67%
Chang YS 2016 (63)	2	RCT	Synbiotics		148/1006	RR	0.44 (0.11, 1.83)	56.7%
Panduru 2015 a (108)	18	RCT	Probiotics		1189/3564	OR	0.64 (0.56, 0.74)	67.04%
Panduru 2015 b (108)	8	RCT	*Lactobacillus* spp.		363/1243	OR	0.70 (0.54, 0.89)	–
Panduru 2015 c (108)	10	RCT	Mixed-strains		826/2321	OR	0.62 (0.52, 0.74)	–
Panduru 2015 d (108)	13	RCT	Probiotics	Prenatal and postpartum	985/2767	OR	0.61 (0.52, 0.71)	–
Panduru 2015 e (108)	4	RCT	Probiotics	Postpartum	115/555	OR	0.95 (0.63, 1.45)	–
Cuello-Garcia 2015 a (80)	15	RCT	Probiotics	Prenatal and postpartum	864/3267	RR	0.71 (0.59, 0.84)	53%
Cuello-Garcia 2015 b (80)	11	RCT	Probiotics	Prenatal and postpartum	423/2777	RR	0.65 (0.55, 0.78)	0%
Cuello-Garcia 2015 c (80)	10	RCT	Probiotics	Prenatal and postpartum	421/1507	RR	0.61 (0.49, 0.76)	37%
Cuello-Garcia 2015 d (80)	7	RCT	Probiotics	Prenatal and postpartum	191/1225	RR	0.63 (0.49, 0.82)	0%
Cuello-Garcia 2015 e (80)	5	RCT	Probiotics	Postpartum	217/790	RR	0.83 (0.58, 1.19)	55%
Cuello-Garcia 2015 f (80)	11	RCT	Probiotics	Prenatal and postpartum	685/2657	RR	0.80 (0.68, 0.93)	32%
Cuello-Garcia 2015 g (80)	2	RCT	Probiotics	Postpartum	51/427	RR	1.67 (0.98, 2.92)	0%
Cuello-Garcia 2015 h (80)	8	RCT	Probiotics	Postpartum	351/2218	RR	0.63 (0.52, 0.77)	0%
Cuello-Garcia 2015 i (80)	16	RCT	Probiotics		953/3509	RR	0.72 (0.61, 0.85)	52%
Cuello-Garcia 2015 j (80)	12	RCT	Probiotics		461/2985	RR	0.68 (0.57, 0.80)	0%
Cuello-Garcia 2015 k (80)	11	RCT	Probiotics		446/1595	RR	0.61 (0.50, 0.74)	30%
Cuello-Garcia 2015 L (80)	16	RCT	Probiotics		902/3447	RR	0.81 (0.70, 0.94)	38%
Cuello-Garcia 2015 m (80)	10	RCT	Probiotics		402/2645	RR	0.72 (0.55, 0.95)	47%
Zuccotti 2015 a (61)	29	RCT	Probiotics		1519/4755	RR	0.78 (0.69, 0.89)	57%
Zuccotti 2015 b (61)	9	RCT	Mixed-strains		350/939	RR	0.54 (0.43, 0.68)	38%
Zuccotti 2015 c (61)	17	RCT	*Lactobacillus* spp.		899/2948	RR	0.90 (0.77, 1.05)	46%
Zuccotti 2015 d (61)	3	RCT	*Bifidobacterium* spp.		270/868	RR	0.89 (0.73, 1.08)	0%
Cao-L 2015 a (45)	6	RCT	Probiotics		769/1955	RR	0.86 (0.77, 0.96)	46%
Cao-L 2015 b (45)	3	RCT	Probiotics	Postpartum	132/484	RR	0.98 (0.73, 1.31)	56%
Cao-L 2015 c (45)	3	RCT	Probiotics	Prenatal and postpartum	637/1471	RR	0.83 (0.74, 0.94)	47%
Mansfield 2014 (109)	27	RCT	Probiotics		2088/6277	RR	0.78 (0.70, 0.88)	59%
Jaramillo 2013 (41)	7	RCT	Probiotics		389/1237	OR	0.64 (0.50, 0.82)	0%
Dang 2013 a (62)	3	RCT	Prebiotics		97/1095	RR	0.80 (0.54, 1.18)	48%
Dang 2013 b (62)	14	RCT	Probiotics		754/2550	RR	0.69 (0.62, 0.78)	57%
Dang 2013 c (62)	7	RCT	*Lactobacillus* spp.		361/1207	RR	0.78 (0.60, 1.01)	53%
Dang 2013 d (62)	2	RCT	*Bifidobacterium* spp.		119/336	RR	0.82 (0.63, 1.07)	0%
Dang 2013 e (62)	6	RCT	mixed-strains		284/1008	RR	0.58 (0.44, 0.76)	43%
Pelucchi 2012 a (110)	13	RCT	Probiotics		930/3092	RR	0.79 (0.71, 0.88)	24%
Pelucchi 2012 b (110)	8	RCT	Probiotics	Prenatal and postpartum	683/2219	RR	0.76 (0.65, 0.89)	31%
Pelucchi 2012 c (110)	4	RCT	Probiotics	postpartum	169/663	RR	0.85 (0.61, 1.19)	32%
Wang-Y 2012 a (111)	8	RCT	Probiotics		208/2290	RD	−0.06(−0.10,-0.03)	0%
Wang-Y 2012 b (111)	8	RCT	Probiotics		355/2097	RD	−0.02(−0.08, −0.03)	56%
Tang-LJ 2012 a (44)	15	RCT	Probiotics		872/3604	RR	0.78 (0.70, 0.88)	30.7%
Tang-LJ 2012 b (44)	9	RCT	Lactobacilli		397/1197	RR	0.70 (0.59, 0.82)	43.7%
Tang-LJ 2012 c (44)	6	RCT	Mixed-strains		500/2415	RR	0.84 (0.72, 0.98)	18.9%
Doege 2012 a (112)	3	RCT	Mixed-strains		514/1956	RR	0.92 (0.83, 1.02)	0%
Doege 2012 b (112)	4	RCT	*Lactobacillus* spp.		228/834	RR	0.82 (0.71, 0.95)	0%
Zhu-DL 2010 a (43)	11	RCT	Probiotics		674/2297	RR	0.80 (0.70, 0.90)	31%
Zhu-DL 2010 b (43)	5	RCT	*Lactobacillus* spp.		238/760	RR	0.85 (0.69, 1.05)	57%
Zhu-DL 2010 c (43)	5	RCT	Mixed-strains		418/1480	RR	0.79 (0.68, 0.93)	0%
Osborn 2009 (113)	2	RCT	Prebiotics		55/432	RR	0.69 (0.40, 1.17)	80%
Lee 2008 a (2)	6	RCT	Probiotics		348/1581	RR	0.69 (0.57, 0.83)	54.8%
Lee 2008 b (2)	5	RCT	Probiotics	Prenatal and postpartum	276/1406	RR	0.61 (0.49, 0.76)	0%
Osborn 2007 a (114)	4	RCT	Probiotics		220/1356	RR	0.80 (0.62, 1.02)	65%
Osborn 2007 b (114)	5	RCT	Probiotics		472/1477	RR	0.82 (0.70, 0.95)	64%
Osborn 2007 c (114)	2	RCT	*Lactobacillus rhamnosus* GG		64/189	RR	0.45 (0.29, 0.72)	0%

**Table 2:** Summary of the meta-analyses of probiotics and adverse events risk.

Study, year (ref)	Number of study	Study design	Exposure	Adverse event	Cases/Total	Type of metrics	Summary effect size (95% CI)	I^2^
Sun-M 2021 (65)	6	RCT	Probiotics	Adverse events	136/1309	RR	1.09 (0.83, 1.44)	0%
Kuang Linghan 2020 a (78)	2	RCT	Probiotics	Death	21/244	RR	0.34 (0.13, 0.91)	0%
Kuang Linghan 2020 b (78)	2	RCT	Probiotics	NEC	32/244	RR	0.38 (0.18, 0.81)	0%
Kuang Linghan 2020 c (78)	2	RCT	Probiotics	Pre-eclampsia	41/322	RR	1.49 (0.85, 2.63)	0%
Cuello-Garcia 2017 a (79)	9	RCT	Prebiotics	Adverse events	951/2876	RR	1.01 (0.92, 1.10)	0%
Cuello-Garcia 2015 b (80)	4	RCT	Probiotics	Adverse events	232/829	RR	1.10 (0.64, 1.91)	51%

### 3.1 Probiotics and AD outcomes

**Figure 3:**
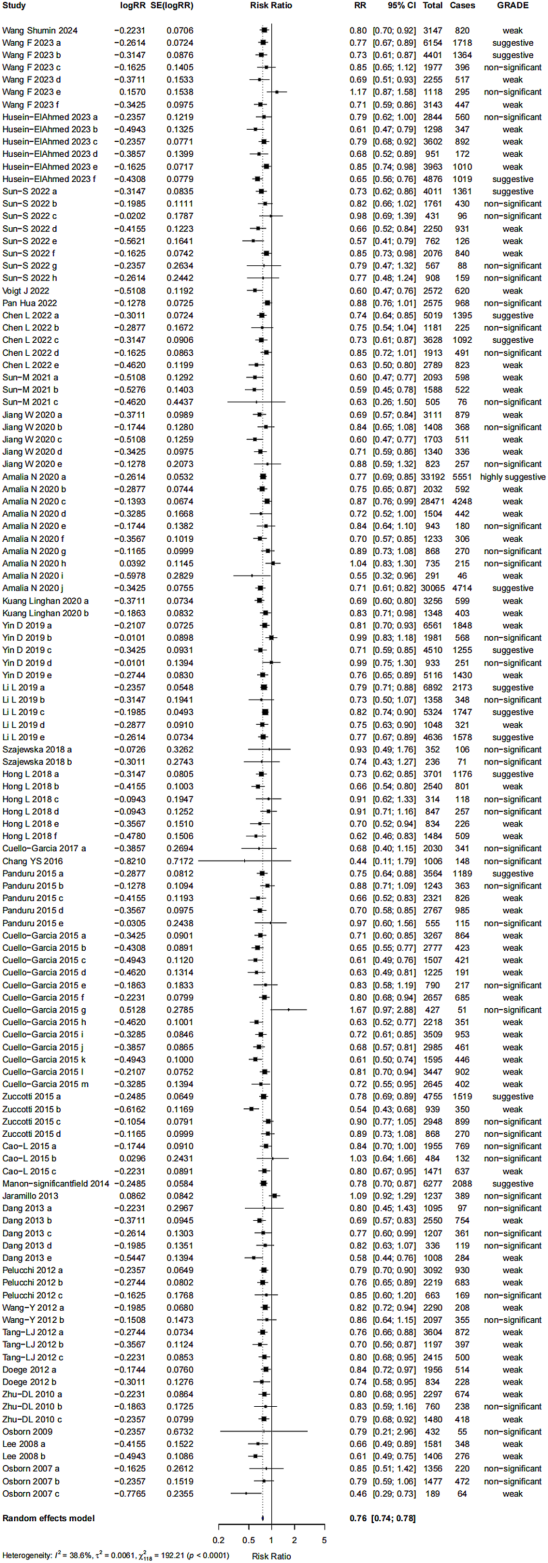
Forest plot of the effect of probiotics on AD risk.

This study found a significant association between probiotics supplementation and the risk of AD (RR = 0.76; 95% CI: 0.74, 0.78; *p* < 0.001) with a low heterogeneity (I^2^ = 0.386, *p* < 0.001) (Figure 3). 11 comparisons (9%) exhibited small-study effect bias, as indicated by an Egger’s asymmetry test with *p* < 0.05. We found that in 58 comparisons, the observed number of studies with significant results exceeded the expected number, suggesting the presence of excess significance bias (Table 3). Among the 119 comparisons, 47 (39%) exhibited heterogeneity (I^2^ > 50%), which may be attributed to variations in probiotics types, timing of interventions, and other contributing factors. Egger’s regression test (*p* = 0.407) showed no evidence of small-study effects, indicating a low likelihood of publication bias. The Trim and Fill analysis further confirmed the robustness of the results (RR = 0.763; 95% CI: 0.745, 0.781), indicating that the current results are relatively consistent.

**Table 3:** Effect estimates, evidence credibility, risk of bias, and heterogeneity assessment in the included meta-analyses.

Study year (ref)	Exposure	Time	RR^a^	*p*-value^b^	I^2^(95% CI)	Q test p-value	Egger’s *p*-value	95%PI	O	E	Excess significance bias *p*-value	RR of the largest study	Evidence credibilit**y**
Wang Shumin (102)	Probiotics		0.80 (0.69, 0.91)	0.00111412	22.5%(0%, 60%)	0.2162	0.053	(0.59, 1.07)	4	0.84	0.001793	0.92 (0.65, 1.32)	Weak
Wang F 2023 a (46)	Probiotics		0.77 (0.67, 0.89)	0.0002946	62.9%(45%, 75%)	0	0.137	(0.44, 1.36)	9	1.65	2.90E-07	0.70 (0.51, 0.96)	Suggestive
Wang F 2023 b (46)	Probiotics	Prenatal and postpartum	0.73 (0.61, 0.86)	0.00027264	70.6%(53%, 82%)	0	0.066	(0.39, 1.34)	7	1.07	6.66E-10	0.95 (0.83, 1.09)	Suggestive
Wang F 2023 c (46)	Probiotics	Postpartum	0.85 (0.64, 1.11)	0.23404639	52.2%(5%, 76%)	0.0217	0.663	(0.39, 1.84)	2	1.34	0.2943	0.79 (0.55, 1.15)	Non-significant
Wang F 2023 d (46)	*Lactobacillus rhamnosus*		0.69 (0.51, 0.93)	0.01552051	72.5%(44%, 87%)	0.0006	0.663	(0.27, 1.76)	4	7.14	0.001341	0.40 (0.30, 0.55)	Weak
Wang F 2023 e (46)	*Lactobacillus* spp.		1.17 (0.87, 1.59)	0.30301001	54.3%(0%, 79%)	0.0323	0.215	(0.49, 2.79)	1	1.07	1	1.27 (0.84, 1.94)	Non-significant
Wang F 2023 f (46)	Mixed probiotics		0.71 (0.58, 0.85)	0.00028342	60.4%(27%, 78%)	0.0025	0.007	(0.43, 1.17)	5	0.78	3.14E-05	0.95 (0.83, 1.09)	Weak
Husein-ElAhmed 2023 a (103)	Probiotics	Postpartum	0.79 (0.62, 1.00)	0.05237969	53.3%(19%, 73%)	0.005	0.034	(0.37, 1.70)	2	1.91	1	0.80 (0.53, 1.19)	Non-significant
Husein-ElAhmed 2023 b (103)	Probiotics	Prenatal and postpartum	0.61 (0.47, 0.79)	0.00018402	44.1%(0%, 76%)	0.0971	0.768	(0.31, 1.19)	5	5.76	0.03075	0.40 (0.27, 0.60)	Weak
Husein-ElAhmed 2023 c (103)	Probiotics	Prenatal and postpartum	0.79 (0.68, 0.92)	0.00168948	38.7%(0%, 67%)	0.0691	0.146	(0.53, 1.18)	6	0.77	2.12E-07	0.96 (0.76, 1.20)	Weak
Husein-ElAhmed 2023 d (103)	Probiotics	Prenatal and postpartum	0.68 (0.52, 0.90)	0.00652819	0%(0%, 79%)	0.533	0.796	(0.43, 1.07)	1	0.71	1	0.76 (0.47, 1.22)	Weak
Husein-ElAhmed 2023 e (103)	Single-strain		0.85 (0.74, 0.98)	0.02925746	41%(3%, 64%)	0.022	0.206	(0.53, 1.37)	5	8.28	0.1891	0.66 (0.46, 0.94)	Weak
Husein-ElAhmed 2023 f (103)	Mixed-strains		0.65 (0.56, 0.76)	7.87E-08	41.6%(1%, 66%)	0.0271	0.022	(0.41, 1.05)	8	1.07	6.88E-13	0.96 (0.76, 1.20)	Suggestive
Sun-S 2022 a (104)	Probiotics		0.73 (0.62, 0.86)	0.00017683	65.6%(43%, 79%)	0.0001	0.079	(0.42, 1.28)	6	1.47	2.55E-07	0.91 (0.78, 1.06)	Suggestive
Sun-S 2022 b (104)	Single-strain		0.82 (0.66, 1.02)	0.07029579	38.2%(0%, 72%)	0.1143	0.922	(0.48, 1.40)	2	0.74	0.2888	0.88 (0.63, 1.22)	Non-significant
Sun-S 2022 c(104)	Single-strain		0.98 (0.69, 1.39)	0.91240937	0%(0%, 90%)	0.9802	0.135	(0.10, 9.54)	0	0.16	NA	0.88 (0.63, 1.22)	Non-significant
Sun-S 2022 d(104)	Mixed-strains		0.66 (0.52, 0.84)	0.00083778	79.3%(60%, 89%)	0	0.016	(0.31, 1.42)	4	0.87	0.001341	0.91 (0.78, 1.06)	Weak
Sun-S 2022 e(104)	Mixed-strains		0.57 (0.41, 0.78)	0.00060358	0%(0%, 90%)	0.7225	0.452	(0.07, 4.66)	2	1.11	0.2207	0.61 (0.41, 0.91)	Weak
Sun-S 2022 e (104)	Probiotics	Prenatal and postpartum	0.85 (0.74, 0.99)	0.03077267	35%(0%, 73%)	0.1611	0.155	(0.61, 1.19)	2	0.78	0.2801	0.91 (0.78, 1.06)	Weak
Sun-S 2022 g (104)	Probiotics	Prenatal and postpartum	0.79 (0.47, 1.32)	0.36812025	45.1%(0%, 84%)	0.162	0.29	(0.00, 124.80)	1	0.15	2.20E-16	0.98 (0.54, 1.80)	Non-significant
Sun-S 2022 h (104)	Probiotics	Postpartum	0.77 (0.48, 1.25)	0.29371811	56.7%(0%, 84%)	0.0554	0.203	(0.17, 3.48)	1	0.54	1	0.80 (0.53, 1.19)	Non-significant
Voigt J 2022 (60)	*Lactobacillus rhamnosus*		0.60 (0.47, 0.75)	9.87E-06	48%(0%, 75%)	0.0439	0.755	(0.32, 1.10)	6	3.94	0.1967	0.64 (0.52, 0.79)	Weak
Pan Hua 2022(105)	Probiotics		0.88 (0.76, 1.01)	0.07507596	39.7%(0%, 73%)	0.1141	0.567	(0.62, 1.24)	2	0.83	0.285	0.91 (0.78, 1.06)	Non-significant
Chen L 2022 a (106)	Probiotics		0.74 (0.64, 0.85)	0.00003473	55.1%(27%, 72%)	0.001	0.119	(0.44, 1.24)	7	3.81	0.09725	0.81 (0.66, 0.99)	Suggestive
Chen L 2022 b (106)	Probiotics	Postpartum	0.75 (0.54, 1.04)	0.08543244	38.1%(0%, 74%)	0.1383	0.016	(0.34, 1.64)	1	0.47	2.20E-16	0.88 (0.57, 1.37)	Non-significant
Chen L 2022 c(106)	Probiotics	Prenatal and postpartum	0.73 (0.61, 0.87)	0.00052046	65%(38%, 40%)	0.0004	0.362	(0.40, 1.34)	6	2.91	0.0507	0.81 (0.66, 0.99)	Suggestive
Chen L 2022 d (106)	*Lactobacillus* spp.		0.85 (0.72, 1.01)	0.06724994	16.2%(0%, 57%)	0.294	0.614	(0.62, 1.17)	1	1.86	0.4292	0.77 (0.54, 1.10)	Non-significant
Chen L 2022 e(106)	Single-strain		0.63 (0.50, 0.80)	0.00010024	68.7%(41%, 83%)	0.0004	0.05	(0.31, 1.30)	6	2.24	0.001766	0.81 (0.66, 0.99)	Weak
Sun-M 2021 a (65)	Probiotics		0.60 (0.47, 0.78)	0.00008495	66.7%(35%, 83%)	0.0014	0.13	(0.27, 1.33)	6	0.56	1.36E-07	1.05 (0.81, 1.37)	Weak
Sun-M 2021 b (65)	Probiotics	Prenatal	0.59 (0.45, 0.78)	0.00026224	70.8%(40%, 86%)	0.0011	0.163	(0.24, 1.44)	5	0.46	2.20E-16	1.05 (0.81, 1.37)	Weak
Sun-M 2021 c (65)	Probiotics	Postpartum	0.63 (0.26, 1.48)	0.28461931			-		1	0.14	2.20E-16	0.88 (0.56, 1.39)	Non-significant
Jiang W 2020 a(64)	Probiotics		0.69 (0.57, 0.84)	0.00019148	66.2%(43%, 80%)	0.0001	0.38	(0.34, 1.41)	8	3.61	0.02092	0.76 (0.56, 1.04)	Weak
Jiang W 2020 b (64)	Single-strain		0.84 (0.66, 1.09)	0.19019584	49.6%(0%, 79%)	0.0642	0.313	(0.42, 1.69)	2	1.09	0.2801	1.25 (0.89, 1.75)	Non-significant
Jiang W 2020 c (64)	Mixed-strains		0.60 (0.47, 0.77)	0.00006072	65.8%(31%, 83%)	0.0029	0.408	(0.28, 1.31)	6	2.15	0.001341	0.76 (0.56, 1.04)	Weak
Jiang W 2020 d (64)	Probiotics	Prenatal and postpartum	0.71 (0.58, 0.85)	0.00034359	15.6%(0%, 59%)	0.3069	0.604	(0.49, 1.01)	4	1.52	0.1025	0.76 (0.56, 1.04)	Weak
Jiang W 2020 e (64)	Probiotics	Postpartum	0.88 (0.59, 1.33)	0.55519065	74.2%(28%, 91%)	0.0088	0.089	(0.15, 5.20)	1	0.75	1	1.25 (0.89, 1.75)	Non-significant
Amalia N 2020 a (48)	Probiotics		0.77 (0.69, 0.85)	4.91E-07	58.1%(38%, 72%)	0	0.002	(0.51, 1.16)	9	2.50	0.0002738	0.93 (0.87, 1.00)	Highly suggestive
Amalia N 2020 b (48)	Probiotics	Prenatal and postpartum	0.75 (0.65, 0.87)	0.00009619	4.5%(0%, 64%)	0.3994	0.083	(0.62, 0.92)	2	1.06	0.2918	0.86 (0.63, 1.16)	Weak
Amalia N 2020 c (48)	Probiotics	Prenatal and postpartum	0.87 (0.76, 0.99)	0.02925746	48.3%(0%, 76%)	0.0508	0.063	(0.63, 1.19)	3	1.09	0.03389	0.93 (0.87, 1.00)	Weak
Amalia N 2020 d (48)	Probiotics	Prenatal and postpartum	0.72 (0.52, 0.99)	0.04883837	74.9%(49%, 88%)	0.0002	0.037	(0.25, 2.08)	4	7.44	0.001341	0.41 (0.27, 0.60)	Weak
Amalia N 2020 e (48)	Probiotics	Postpartum	0.84 (0.64, 1.10)	0.20408463	7%(0%, 86%)	0.3575	0.112	(0.44, 1.62)	0	0.48	NA	1.22 (0.85, 1.76)	Non-significant
Amalia N 2020 f (48)	*L. rhamnosus* HN001		0.70 (0.57, 0.85)	0.00034359	0%(0%, 85%)	0.56	0.562	(0.45, 1.08)	2	1.72	1	0.69 (0.49, 0.98)	Weak
Amalia N 2020 g(48)	Bifidbacterium animalis HN019		0.89 (0.73, 1.08)	0.23013934	0%(0%, 90%)	0.9512	0.507	(0.25, 3.18)	0	0.43	NA	0.86 (0.63, 1.16)	Non-significant
Amalia N 2020 h (48)	*Lactobacillus rhamnosus* GG		1.04 (0.83, 1.30)	0.71884713	0%(0%, 79%)	0.5445	0.206	(0.72, 1.50)	0	0.40	NA	0.88 (0.63, 1.22)	Non-significant
Amalia N 2020 i (48)	*L. paracasei* F19		0.55 (0.32, 0.97)	0.03939854			–		0	0.82	0.1573	0.49 (0.24, 1.02)	Weak
Amalia N 2020 j (48)	Mixed-strains		0.71 (0.61, 0.82)	6.48E-06	71.1%(53%, 82%)	0	0.001	(0.42, 1.19)	7	1.63	0.0001768	0.93 (0.87, 1.00)	Suggestive
Kuang Linghan 2020 a (78)	Probiotics		0.69 (0.60, 0.80)	9.11E-07	0%(0%, 65%)	0.7672	0.996	(0.58, 0.83)	4	3.03	0.4795	0.70 (0.51, 0.96)	Weak
Kuang Linghan 2020 b (78)	Probiotics		0.83 (0.70, 0.97)	0.02144822	0%(0%, 79%)	0.4779	0.733	(0.64, 1.08)	1	1.35	1	0.78 (0.59, 1.02)	Weak
Yin D 2019 a (42)	Probiotics		0.81 (0.70, 0.93)	0.00317774	64.9%(45%, 78%)	0	0.496	(0.47, 1.40)	7	5.89	0.6321	0.78 (0.62, 0.98)	Weak
Yin D 2019 b (42)	*Lactobacillus* spp.		0.99 (0.83, 1.18)	0.91240937	27.6%(0%, 65%)	0.1901	0.701	(0.67, 1.46)	0	1.38	0.2918	0.83 (0.65, 1.05)	Non-significant
Yin D 2019 c (42)	Mixed-strains		0.71 (0.59, 0.85)	0.00025222	71%(46%, 84%)	0.0002	0.71	(0.39, 1.30)	7	3.74	0.06006	0.78 (0.62, 0.98)	Suggestive
Yin D 2019 d (42)	Probiotics	Postpartum	0.99 (0.77, 1.33)	0.92828721	44.2%(0%, 78%)	0.1103	0.674	(0.45, 2.18)	0	0.88	0.2733	1.27 (0.84, 1.94)	Non-significant
Yin D 2019 e (42)	Probiotics	Prenatal and postpartum	0.76 (0.65, 0.90)	0.00168948	68.9%(47%, 82%)	0	0.826	(0.42, 1.38)	7	4.44	0.07984	0.78 (0.62, 0.98)	Weak
Li L 2019 a (59)	Probiotics		0.79 (0.71, 0.88)	0.00002554	52.5%(27%, 69%)	0.0007	0.289	(0.52, 1.20)	8	2.30	1.07E-05	0.91 (0.78, 1.06)	Suggestive
Li L 2019 b (59)	Probiotics	Postpartum	0.73 (0.50, 1.07)	0.10959858	73.7%(46%, 87%)	0.0004	0.629	(0.22, 2.38)	1	5.99	4.46E-05	0.40 (0.30, 0.55)	Non-significant
Li L 2019 c (59)	Probiotics	Prenatal and postpartum	0.82 (0.75, 0.91)	0.00011812	29%(0%, 59%)	0.1156	0.201	(0.64, 1.06)	7	1.68	0.0001857	0.91 (0.78, 1.06)	Suggestive
Li L 2019 d (59)	*L. rhamnosus*		0.75 (0.63, 0.90)	0.0016327	0%(0%, 75%)	0.4395	0.264	(0.58, 0.97)	2	1.27	0.2733	0.77 (0.54, 1.10)	Weak
Li L 2019 e (59)	Mixed-strains		0.77 (0.66, 0.88)	0.03%	62.4%(34%, 78%)	0.0007	0.139	(0.48, 1.23)	5	1.43	3.47E-05	0.91 (0.78, 1.06)	Suggestive
Szajewska 2018 a (107)	*Lactobacillus rhamnosus* GG	Prenatal and postpartum	0.93 (0.49, 1.76)	0.82587115	71.2%(0%, 92%)	0.0276	0.41	(0.00, 1412.82)	1	1.83	0.2207	0.51 (0.31, 0.86)	Non-significant
Szajewska 2018 b (107)	*Lactobacillus rhamnosus* GG	Prenatal and postpartum	0.74 (0.43, 1.26)	0.25847622			–		1	0.98	1	0.57 (0.33, 0.97)	Non-significant
Hong L 2018 a (47)	Probiotics		0.73 (0.62, 0.85)	0.00006607	61.1%(37%, 76%)	0.0002	0.201	(0.41, 1.31)	5	1.13	4.06E-05	1.05 (0.80, 1.37)	Suggestive
Hong L 2018 b (47)	Probiotics	Prenatal and postpartum	0.66 (0.54, 0.80)	0.00003473	63.6%(34%, 80%)	0.001	0.357	(0.35, 1.27)	5	0.74	3.14E-05	1.05 (0.80, 1.37)	Weak
Hong L 2018 c (47)	Probiotics	Prenatal	0.91 (0.62, 1.33)	0.61707508			–		0	0.48	NA	0.77 (0.54, 1.10)	Non-significant
Hong L 2018 d (47)	Probiotics	Postpartum	0.91 (0.71, 1.16)	0.4237108	27.2%(0%, 71%)	0.2397	0.076	(0.51, 1.63)	0	0.47	NA	1.15 (0.85, 1.55)	Non-significant
Hong L 2018 e (47)	*Lactobacillus* spp.		0.70 (0.52, 0.94)	0.01778809	38.3%(0%, 77%)	0.1654	0.441	(0.31, 1.58)	2	0.26	2.20E-16	1.04 (0.71, 1.54)	Weak
Hong L 2018 f (47)	Mixed-strains		0.62 (0.46, 0.83)	0.00168948	77.5%(53%, 89%)	0.0002	0.32	(0.23, 1.65)	4	0.41	2.20E-16	1.05 (0.80, 1.37)	Weak
Cuello-Garcia 2017 a (79)	Prebiotics		0.68 (0.40, 1.15)	0.1498674	66.7%(21%, 86%)	0.0103	0.255	(0.16, 2.99)	3	0.30	NA	1.02 (0.82, 1.27)	Non-significant
Chang YS (63)	Synbiotics		0.44 (0.11, 1.83)	0.25847622			–		1	0.67	1	0.70 (0.51, 0.96)	Non-significant
Panduru 2015 a (108)	Probiotics		0.75 (0.64, 0.88)	0.00041556	57.8%(29%, 75%)	0.0012	0.421	(0.43, 1.29)	4	1.39	0.002022	0.91 (0.78, 1.06)	Suggestive
Panduru 2015 b (108)	*Lactobacillus* spp.		0.88 (0.71, 1.09)	0.22627889	29.1%(0%, 68%)	0.1955	0.19	(0.55, 1.41)	1	0.65	1	0.88 (0.63, 1.22)	Non-significant
Panduru 2015 c (108)	Mixed-strains		0.66 (0.52, 0.83)	0.00040013	69.4%(41%, 84%)	0.0006	0.08	(0.32, 1.35)	3	0.85	0.03501	0.91 (0.78, 1.06)	Weak
Panduru 2015 d (108)	Probiotics	Prenatal and postpartum	0.70 (0.58, 0.85)	0.00022425	63.4%(33%, 80%)	0.0011	0.201	(0.38, 1.28)	4	1.07	0.001793	0.91 (0.78, 1.06)	Weak
Panduru 2015 e (108)	Probiotics	postpartum	0.97 (0.60, 1.56)	0.89656643	40.7%(0%, 80%)	0.1675	0.854	(0.18, 5.25)	0	0.27	NA	0.88 (0.56, 1.39)	Non-significant
Cuello-Garcia 2015 a (80)	Probiotics	Prenatal and postpartum	0.71 (0.59, 0.84)	0.00010446	53.4%(16%, 74%)	0.0076	0.117	(0.40, 1.23)	7	0.83	5.28E-10	1.05 (0.81, 1.36)	Weak
Cuello-Garcia 2015 b (80)	Probiotics	Prenatal and postpartum	0.65 (0.55, 0.78)	3.01E-06	0%(0%, 60%)	0.7525	0.815	(0.53, 0.80)	4	2.34	0.1179	0.70 (0.51, 0.96)	Weak
Cuello-Garcia 2015 c (80)	Probiotics	Prenatal and postpartum	0.61 (0.49, 0.76)	8.59E-06	37%(0%, 70%)	0.1127	0.825	(0.36, 1.05)	6	8.30	0.1138	0.41 (0.27, 0.60)	Weak
Cuello-Garcia 2015 d (80)	Probiotics	Prenatal and postpartum	0.63 (0.49, 0.82)	0.00060358	0%(0%, 71%)	0.7846	0.963	(0.45, 0.89)	2	3.18	0.445	0.514 (0.31, 0.86)	Weak
Cuello-Garcia 2015 e (80)	Probiotics	Postpartum	0.83 (0.58, 1.19)	0.30772846	55.2%(0%, 83%)	0.0631	0.004	(0.29, 2.41)	1	0.34	2.20E-16	1.10 (0.77, 1.58)	Non-significant
Cuello-Garcia 2015 f (80)	Probiotics	Prenatal and postpartum	0.80 (0.68, 0.93)	0.00495415	32.2%(0%, 67%)	0.1414	0.274	(0.54, 1.17)	4	0.61	0.001653	1.05 (0.81, 1.36)	Weak
Cuello-Garcia 2015 g (80)	Probiotics	Postpartum	1.67 (0.98, 2.92)	0.05743312			–		0	0.74	0.1573	1.87 (1.00, 3.52)	Non-significant
Cuello-Garcia 2015 h (80)	Probiotics	Postpartum	0.63 (0.52, 0.77)	3.66E-06	0%(0%, 68%)	0.6526	0.612	(0.49, 0.80)	4	1.87	0.1025	0.70 (0.51, 0.96)	Weak
Cuello-Garcia 2015 i (80)	Probiotics		0.72 (0.61, 0.85)	0.00007815	51.6%(14%, 73%)	0.0088	0.117	(0.43, 1.21)	7	0.89	5.76E-10	1.05 (0.81, 1.36)	Weak
Cuello-Garcia 2015 j (80)	Probiotics		0.68 (0.57, 0.80)	6.18E-06	0%(0%, 58%)	0.7016	0.858	(0.56, 0.82)	4	2.57	0.505	0.70 (0.51, 0.96)	Weak
Cuello-Garcia 2015 k (80)	Probiotics		0.61 (0.50, 0.74)	9.11E-07	30.1%(0%, 66%)	0.1593	0.849	(0.38, 0.97)	6	9.05	0.01902	0.41 (0.27, 0.60)	Weak
Cuello-Garcia 2015 l (80)	Probiotics		0.81 (0.70, 0.94)	0.00560563	38.3%(0%, 66%)	0.06	0.025	(0.54, 1.23)	5	0.89	3.61E-05	1.05 (0.81, 1.36)	Weak
Cuello-Garcia 2015 m (80)	Probiotics		0.72 (0.55, 0.95)	0.02144822	47%(0%, 74%)	0.049	0.721	(0.34, 1.53)	4	2.18	0.1138	0.70 (0.51, 0.96)	Weak
Zuccotti 2015 a (61)	Probiotics		0.78 (0.69, 0.89)	0.00026224	56.7%(35%, 71%)	0.0001	0.347	(0.45, 1.36)	7	3.01	0.01473	0.86 (0.63, 1.16)	Suggestive
Zuccotti 2015 b (61)	Mixed-strains		0.54 (0.43, 0.68)	9.30E-08	38%(0%, 71%)	0.1168	0.311	(0.32, 0.92)	3	1.44	0.03389	0.79 (0.57, 1.08)	Weak
Zuccotti 2015 c (61)	*Lactobacillus* spp.		0.90 (0.77, 1.05)	0.18024534	46%(5%, 69%)	0.0202	0.932	(0.55, 1.46)	4	1.46	0.001986	0.88 (0.63, 1.22)	Non-significant
Zuccotti 2015 d (61)	*Bifidobacterium* spp.		0.89 (0.73, 1.08)	0.23013934	0%(0%, 90%)	0.9512	0.507	(0.25, 3.18)	0	0.43	NA	0.86 (0.63, 1.16)	Non-significant
Cao-L 2015 a. (45)	Probiotics		0.84 (0.70, 1.00)	0.05237969	45.6%(0%, 78%)	0.1013	0.694	(0.53, 1.33)	2	0.65	0.2733	0.91 (0.78, 1.06)	Non-significant
Cao-L 2015 b (45)	Probiotics	Postpartum	1.03 (0.64, 1.66)	0.89656643	56.2%(0%, 87%)	0.1023	0.306	(0.01, 157.20)	2	0.45	2.20E-16	0.91 (0.78, 1.06)	Non-significant
Cao-L 2015 c (45)	Probiotics	Prenatal and postpartum	0.80 (0.67, 0.95)	0.01313824	46.8%(0%, 84%)	0.1526	0.287	(0.14, 4.59)	0	0.42	NA	0.80 (0.53, 1.19)	Weak
Mansfield 2014 (109)	Probiotics		0.78 (0.70, 0.88)	0.00006892	59.3%(38%, 73%)	0	0.334	(0.49, 1.27)	10	2.22	4.13E-09	0.91 (0.78, 1.06)	Suggestive
Jaramillo 2013 (41)	Probiotics		1.09 (0.92, 1.28)	0.32708612	0%(0%, 71%)	0.5109	0.602	(0.87, 1.35)	0	2.18	0.09426	0.72 (0.50, 1.03)	Non-significant
Dang 2013 a (62)	Prebiotics		0.80 (0.45, 1.44)	0.45929999	48.4%(0%, 85%)	0.1444	0.693	(0.00, 291.62)	1	0.28	2.20E-16	0.81 (0.46, 1.40)	Non-significant
Dang 2013 b (62)	Probiotics		0.69 (0.58, 0.84)	0.00011339	57%(22%, 76%)	0.0044	0.965	(0.38, 1.26)	5	12.14	8.98E-08	0.40 (0.30, 0.55)	Weak
Dang 2013 c (62)	*Lactobacillus* spp.		0.77 (0.60, 1.00)	0.05237969	52.1%(0%, 80%)	0.0511	0.199	(0.38, 1.58)	2	1.43	0.2801	0.77 (0.54, 1.10)	Non-significant
Dang 2013 d (62)	*Bifidobacterium* spp.		0.82 (0.63, 1.07)	0.13887325			–		0	0.49	NA	0.79 (0.57, 1.08)	Non-significant
Dang 2013 e (62)	Mixed-strains		0.58 (0.44, 0.76)	0.00007815	42.6%(0%, 77%)	0.1212	0.429	(0.29, 1.14)	3	4.78	0.02846	0.40 (0.30, 0.55)	Weak
Pelucchi 2012 a (110)	Probiotics		0.79 (0.69, 0.89)	0.0002156	22.5%(0%, 60%)	0.2162	0.394	(0.60, 1.04)	4	2.54	0.5104	0.81 (0.66, 0.99)	Weak
Pelucchi 2012 b (110)	Probiotics	Prenatal and postpartum	0.76 (0.65, 0.89)	0.00067386	28.9%(0%, 68%)	0.1972	0.644	(0.54, 1.08)	4	1.79	0.1025	0.81 (0.66, 0.99)	Weak
Pelucchi 2012 c (110)	Probiotics	Postpartum	0.85 (0.60, 1.20)	0.35757276	32.7%(0%, 76%)	0.2159	0.159	(0.28, 2.57)	0	0.27	NA	1.10 (0.77, 1.58)	Non-significant
Wang-Y 2012 a (111)	Probiotics		0.82 (0.72, 0.94)	0.00480236	17%(0%, 60%)	0.2954	0.641	(0.63, 1.07)	2	1.88	1	0.81 (0.66, 0.99)	Weak
Wang-Y 2012 b (111)	Probiotics		0.86 (0.64, 1.14)	0.29371811	47.3%(0%, 77%)	0.0632	0.241	(0.40, 1.84)	3	1.93	0.4142	0.70 (0.51, 0.96)	Non-significant
Wang-Y 2012 b (44)	Probiotics		0.76 (0.66, 0.88)	0.00016991	26%(0%, 60%)	0.1676	0.184	(0.54, 1.06)	5	1.06	3.47E-05	0.91 (0.72, 1.14)	Weak
Tang-LJ 2012 b (44)	Lactobacilli		0.70 (0.56, 0.87)	0.0016327	40.8%(0%, 73%)	0.0955	0.102	(0.40, 1.23)	4	1.65	0.1088	0.77 (0.54, 1.10)	Weak
Tang-LJ 2012 c (44)	Mixed-strains		0.80 (0.68, 0.95)	0.01108525	9%(0%, 77%)	0.3579	0.896	(0.60, 1.08)	2	0.48	2.20E-16	0.91 (0.72, 1.14)	Weak
Doege 2012 a (112)	Mixed-strains		0.84 (0.72, 0.97)	0.02088815	0%(0%, 90%)	0.4653	0.501	(0.32, 2.20)	1	1.10	1	0.81 (0.66, 0.99)	Weak
Doege 2012 b (112)	*Lactobacillus* spp.		0.74 (0.57, 0.94)	0.01595252	21%(0%, 88%)	0.2839	0.607	(0.35, 1.55)	1	1.29	1	0.72 (0.50, 1.02)	Weak
Zhu-DL 2010 a (43)	Probiotics		0.80 (0.67, 0.94)	0.00853849	31.3%(0%, 66%)	0.1493	0.163	(0.54, 1.18)	3	1.91	0.4344	0.81 (0.66, 0.99)	Weak
Zhu-DL 2010 b (43)	*Lactobacillus* spp.		0.83 (0.59, 1.16)	0.27571314	56.8%(0%, 84%)	0.0549	0.234	(0.29, 2.37)	1	0.35	2.20E-16	1.10 (0.77, 1.58)	Non-significant
Zhu-DL 2010 c (43)	Mixed-strains		0.79 (0.68, 0.93)	0.00385242	0%(0%, 79%)	0.899	0.235	(0.62, 1.02)	1	1.11	1	0.81 (0.66, 0.99)	Weak
Osborn 2009 (113)	Prebiotics		0.79 (0.21, 2.94)	0.7263387			–		1	1.09	1	0.42 (0.21, 0.84)	Non-significant
Lee 2008 a (2)	Probiotics		0.66 (0.49, 0.89)	0.00672832	54.8%(0%, 82%)	0.0514	0.119	(0.28, 1.54)	4	1.94	0.08326	0.70 (0.51, 0.96)	Weak
Lee 2008 b (2)	Probiotics	Prenatal and postpartum	0.61 (0.49, 0.75)	6.18E-06	0%(0%, 79%)	0.5662	0.033	(0.43, 0.86)	4	1.44	0.0007962	0.70 (0.51, 0.96)	Weak
Osborn 2007 a (114)	Probiotics		0.85 (0.51, 1.42)	0.54186181	65.1%(0%, 88%)	0.0353	0.75	(0.11, 6.85)	1	1.09	1	0.70 (0.51, 0.96)	Non-significant
Osborn 2007 b (114)	Probiotics		0.79 (0.59, 1.07)	0.12851098	63.6%(4%, 86%)	0.0268	0.451	(0.31, 2.05)	3	1.17	0.02535	0.81 (0.66, 0.99)	Non-significant
Osborn 2007 c (114)	*Lactobacillus rhamnosus* GG		0.46 (0.29, 0.73)	0.00093296			-		2	0.97	0.1573	0.51 (0.31, 0.86)	Weak

a: RR of random-effects model; b: p value of random-effects model; O: the observed number of studies; E: the expected number.

In terms of the level of evidence, associations are classified into five categories: convincing, highly suggestive, suggestive, weak and non-significant (Table 3). The evaluation of one meta-analysis provided evidence at the “highly suggestive” level, indicating a negative association between probiotics supplementation and the risk of AD (RR: 0.77, 95% CI: 0.69–0.85) (48). No associations were identified at the “convincing” level of evidence in this study. This study found that 15 comparisons (13%) provided evidence classified as “suggestive” while 60 (50%) were classified as having “weak” evidence. The remaining 43 (36%) comparisons were classified as providing “non-significant” evidence. Among them, the 95% prediction intervals of 10 comparisons did not include the null value of 1. When applying a significance threshold of *p* < 0.05, 80 out of 119 comparisons (67%) demonstrated statistical significance under the random-effects model. When the threshold was set at *p* < 0.001, 49 comparisons (41%) remained statistically significant. At a more stringent threshold of *p* < 0.000001, only 6 comparisons retained statistical significance under the random-effects model.

### 3.2 Different type of probiotics and AD outcomes

#### 3.2.1 *Lactobacillus* spp

**Figure 4:**
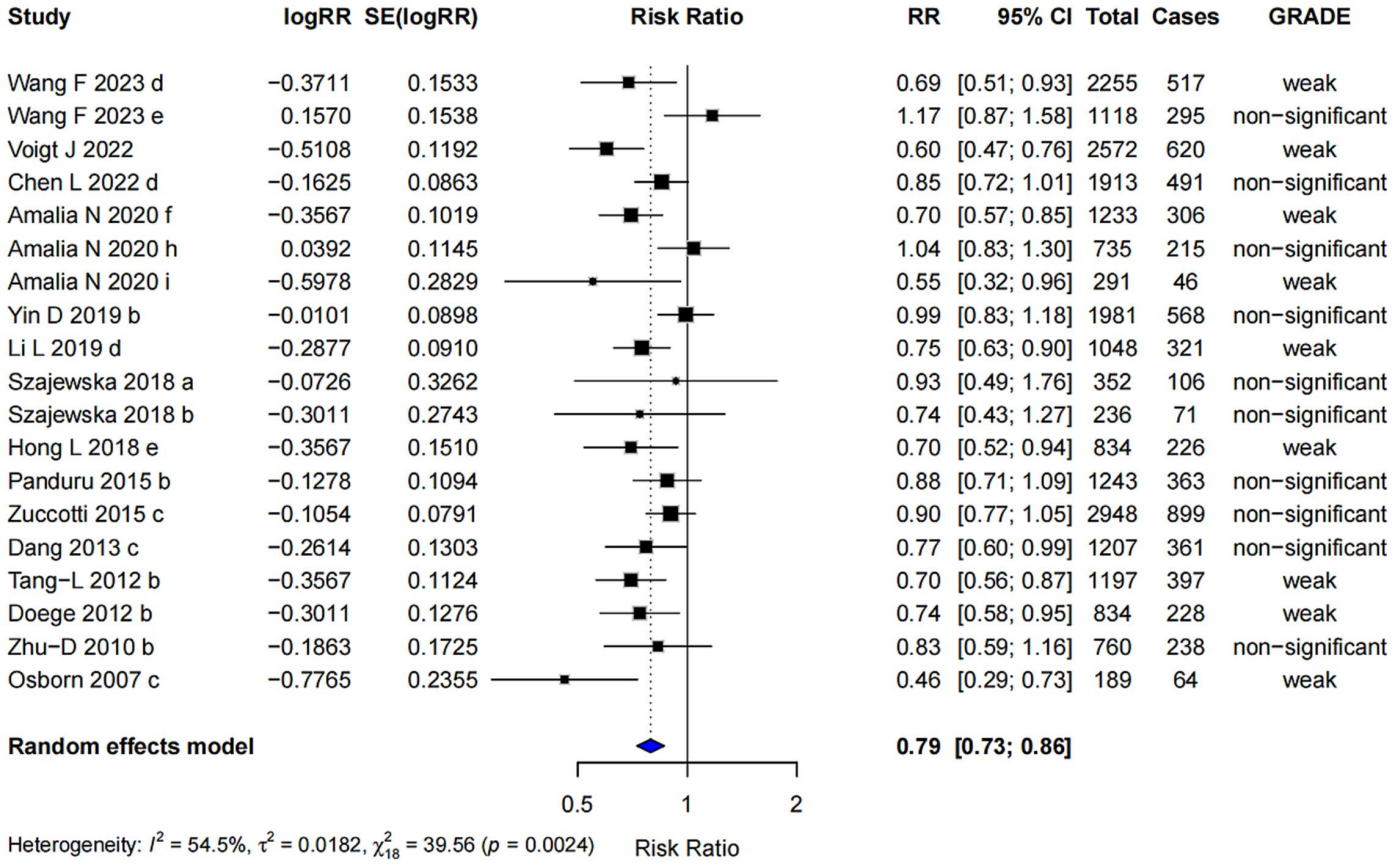
Subgroup analysis of the effect of *Lactobacillus* spp. on AD risk.

In the subgroup analysis, *Lactobacillus* spp. supplementation was associated with a reduced risk of AD (RR = 0.79; 95% CI: 0.73, 0.86). The highest level of evidence achieved was classified as “weak” (Figure 4). Egger’s regression test yielded a *p*-value of 0.258, indicating no evidence of small-study effects. Furthermore, the Trim and Fill analysis showed a robust pooled effect estimate, suggesting that the results are relatively stable.

#### 3.2.2 *Bifidobacterium* spp

**Figure 5:**
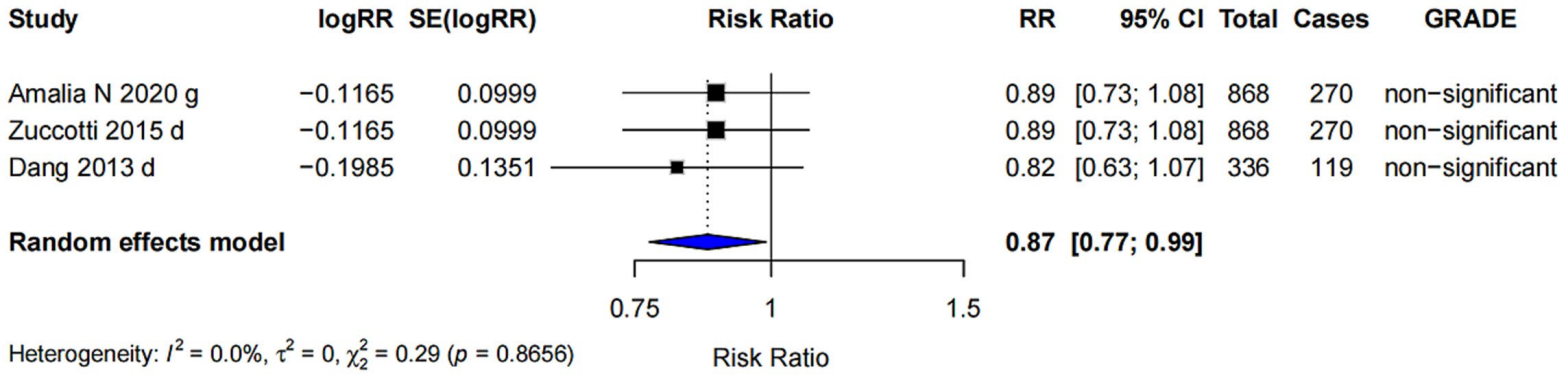
Subgroup analysis of the effect of *Bifidobacterium* spp. on AD risk.

For *Bifidobacterium* spp., the pooled effect size was 0.87 (95% CI: 0.77, 0.99) (Figure 5). However, the strength of evidence was rated as “non-significant” and no further bias assessments were conducted due to the limited number of comparisons available.

#### 3.2.3 Single-strain probiotics

**Figure 6:**
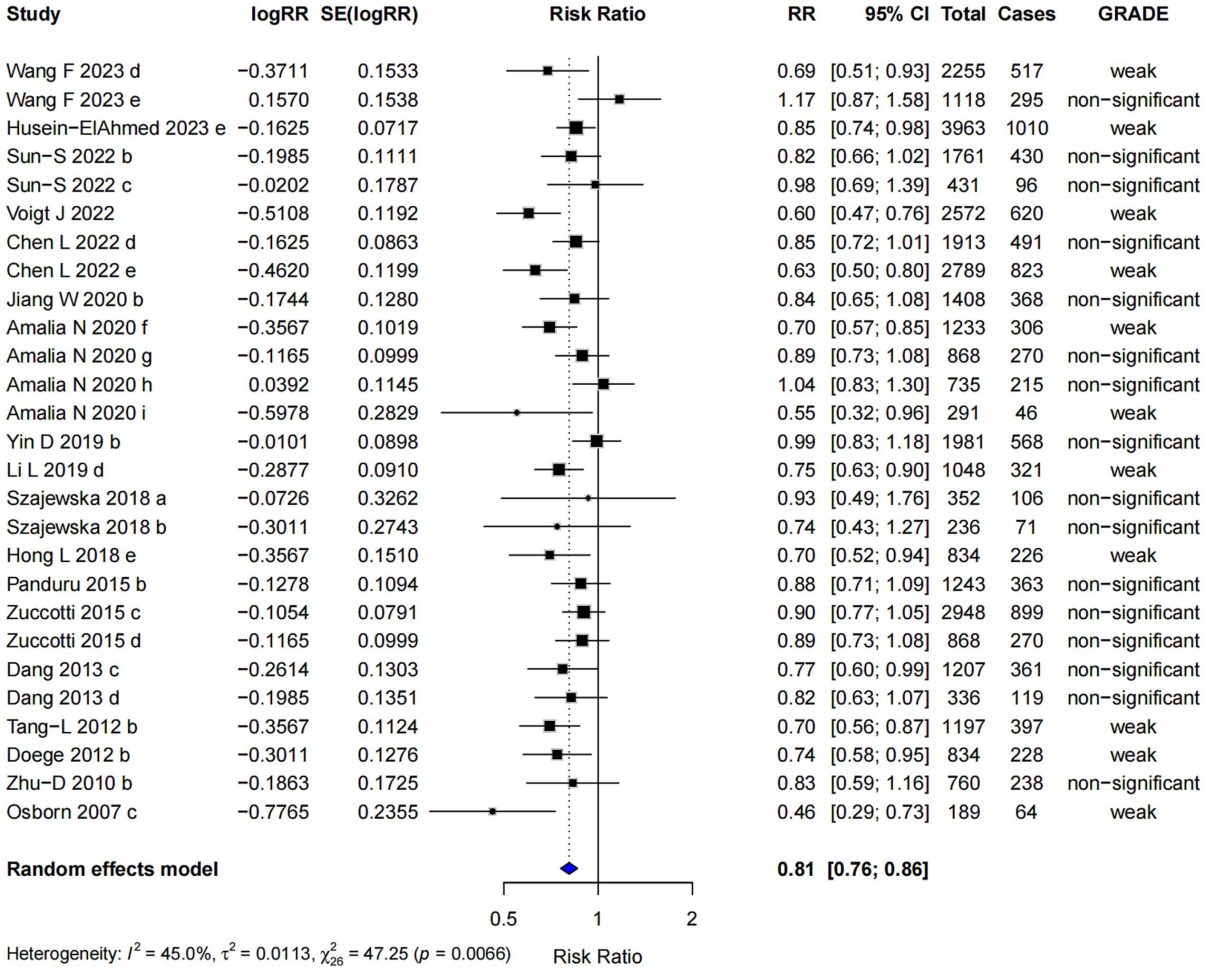
Subgroup analysis of the effect of single-strain probiotics on AD risk.

Single-strain probiotics were associated with a reduced risk of AD (RR = 0.81; 95% CI: 0.76, 0.86), with the highest level of evidence classified as “weak” (Figure 6). Egger’s regression test (*p* = 0.226) indicated no evidence of publication bias. The robustness of the pooled estimate was supported by Trim and Fill analysis, suggesting consistency in the observed association.

#### 3.2.4 Mixed-strains probiotics

**Figure 7:**
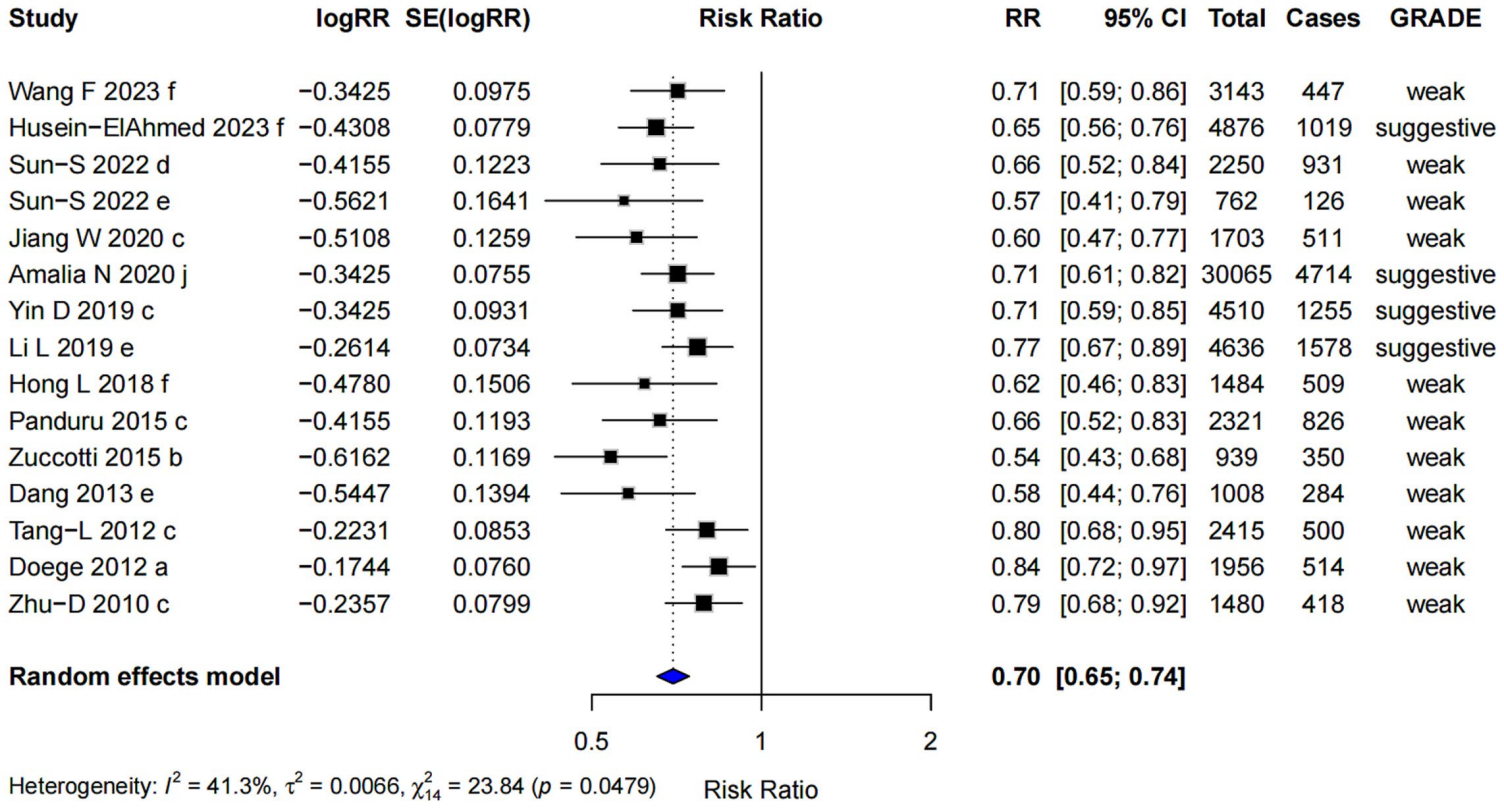
Subgroup analysis of the effect of mixed-strains probiotics on AD risk.

Mixed-strains probiotics showed the most pronounced association with a lower risk of AD among all subgroups (RR = 0.70; 95% CI: 0.65, 0.74), with the highest evidence level graded as “suggestive” (Figure 7). However, Egger’s regression test indicated the presence of small-study effects (*p* = 0.002), suggesting potential publication bias. Despite this, the Trim and Fill analysis produced similar results, indicating that the observed effect estimate was relatively robust after adjustment.

#### 3.2.5 Prebiotics and synbiotics

Current evidence does not provide strong support for an association between prebiotics (RR = 0.69; 95% CI: 0.43, 1.13) or synbiotics (RR = 0.44; 95% CI: 0.11, 1.79) and reduced risk of AD. Both interventions showed wide confidence intervals and limited statistical precision, making it difficult to draw definitive conclusions. Further high-quality studies are warranted to better understand their potential roles in the prevention of AD.

### 3.3 Supplement time of probiotics and AD outcomes

#### 3.3.1 Prenatal probiotics supplementation

**Figure 8:**
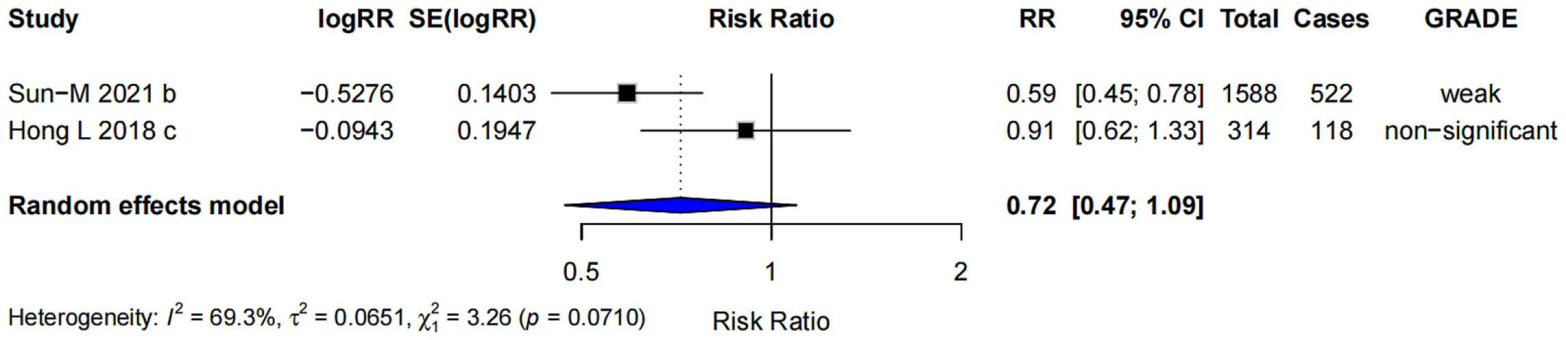
Subgroup analysis of the effect of prenatal supplementation of probiotics on AD risk.

The subgroup analysis for prenatal probiotics supplementation only (RR = 0.72; 95% CI: 0.47, 1.09) (Figure 8) showed no strong evidence to support a significant effect on the risk of AD. The confidence interval includes 1.0, suggesting that the effect is uncertain and not statistically significant.

#### 3.3.2 Postnatal probiotics supplementation

**Figure 9:**
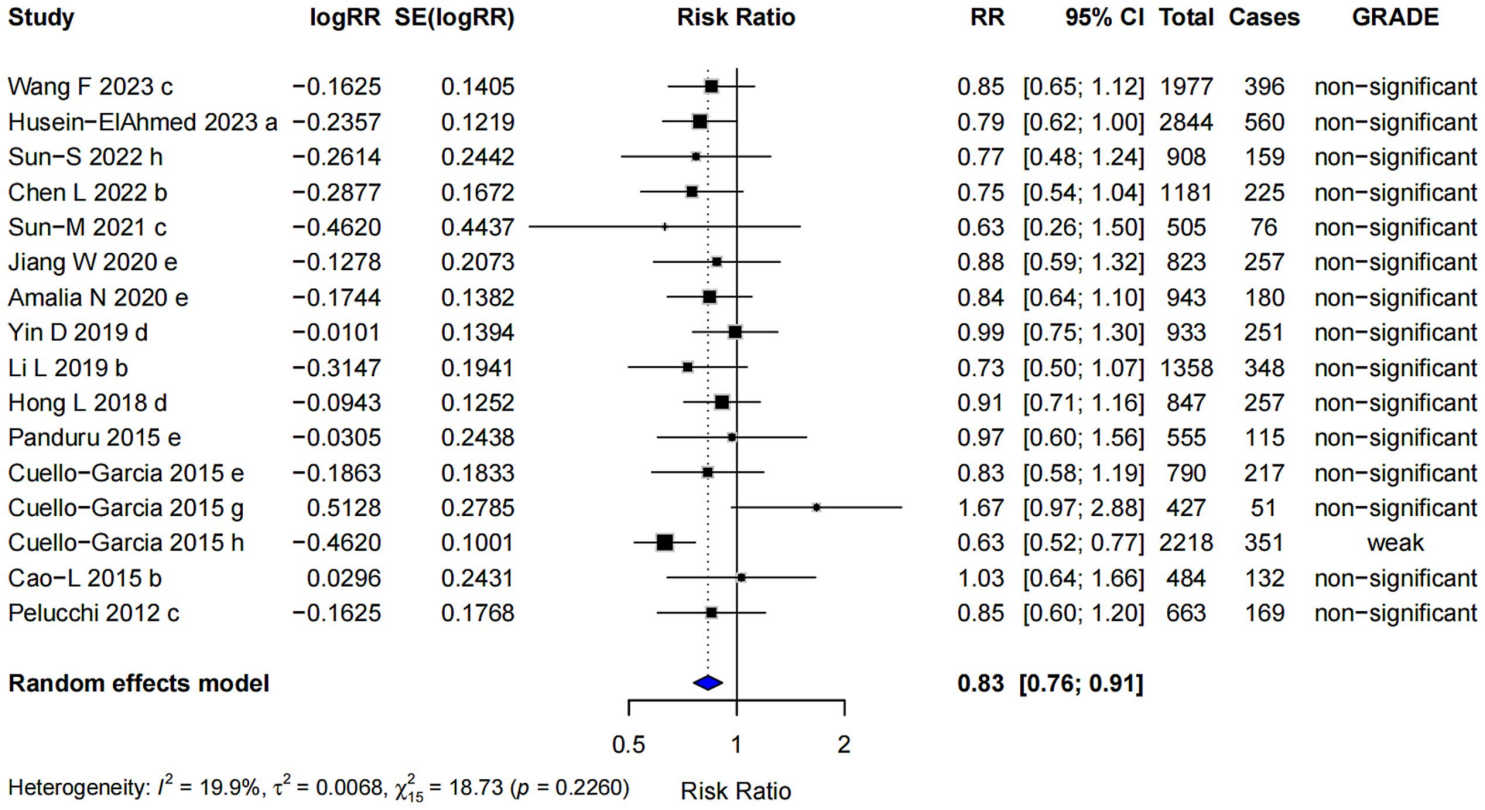
Subgroup analysis of the effect of postpartum supplementation of probiotics on AD risk.

The pooled risk ratio for postnatal probiotics supplementation alone was RR = 0.83 (95% CI: 0.76, 0.91). However, nearly all of the evidence was classified as “non-significant” (Figure 9), indicating that the observed effect was insufficient to draw definitive conclusions about its efficacy in reducing the risk of AD. In this subgroup, Egger’s regression test yielded a *p*-value of 0.382, suggesting no significant evidence of publication bias, indicating that the observed results are unlikely to be influenced by selective reporting of studies.

#### 3.3.3 Combined prenatal and postnatal probiotics supplementation

**Figure 10:**
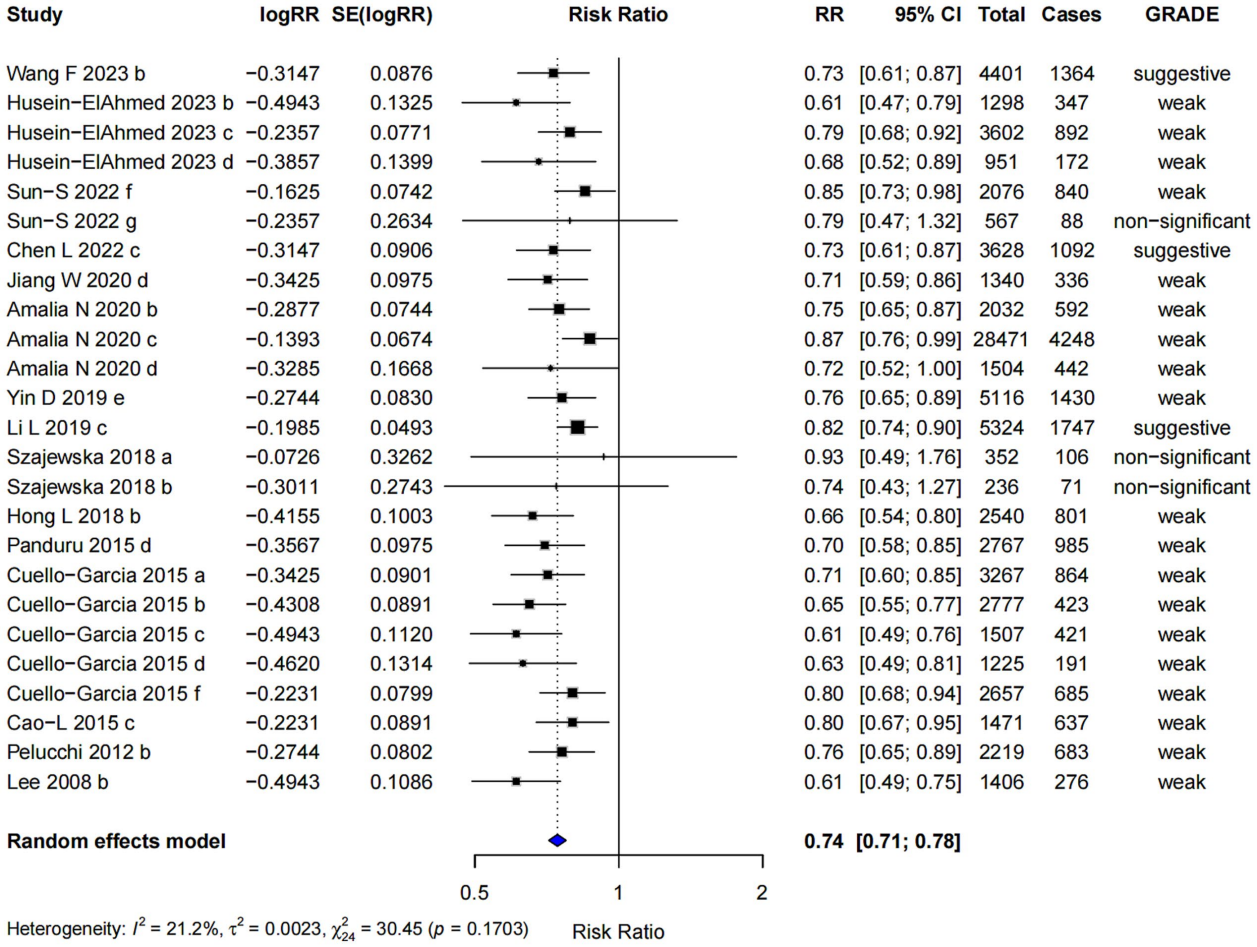
Subgroup analysis of the effect of combined prenatal and postpartum supplementation of probiotics on AD risk.

In the analysis of combined prenatal and postnatal probiotics supplementation (RR = 0.74; 95% CI: 0.71, 0.78), the intervention was associated with a reduced risk of AD, and the highest evidence level was classified as “suggestive” (Figure 10). Egger’s regression test for small-study effects in this subgroup yielded a p-value of 0.02, suggesting the presence of a small-study effect, which may indicate some degree of bias in the included studies. Despite this, the trim and fill analysis demonstrated the robustness of the combined effect size, indicating that the overall effect was not significantly altered by the potential small-study bias.

The summary of evidence levels for probiotics strain/intervention timing and the risk of AD is presented in Table 4.

**Table 4:** Summary of evidence levels for probiotic strain/intervention timing and risk of AD.

Evidence level(n)Subgroup		Non-significant	Weak	Suggestive	Highly suggestive	Convincing	Re-evaluated evidence level
Probiotic strains	*Lactobacillus* spp.	10	9	0	0	0	Weak
	*Bifidobacterium* spp.	3	0	0	0	0	Non-significant
Strain numbers	Mixed-strains	0	11	4	0	0	Highly suggestive
	Single-strain	16	11	0	0	0	Suggestive
Timing	Prenatal	1	1	0	0	0	Weak
	Postpartum	15	1	0	0	0	Suggestive
	Prenatal and postpartum	3	19	3	0	0	Highly suggestive

Re-evaluated evidence level: evidence levels obtained after reanalysis of the original studies.

### 3.4 Probiotics and adverse events outcomes

**Figure 11:**
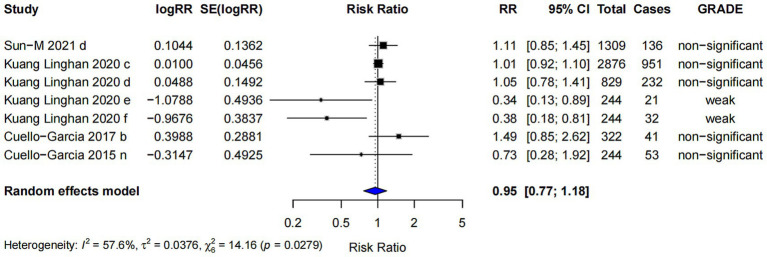
Forest plot between probiotics and adverse events.

This study also found that probiotics supplementation did not increase the risk of adverse events (RR = 0.95; 95% CI: 0.77, 1.18; *p* < 0.001), and the highest evidence level was classified as “weak” (Figure 11). The studies on the incidence of adverse reactions related to probiotics are presented in Table 2.

### 3.5 Different type of study designs and AD outcomes

In the stratified analysis by study design, the pooled relative risk derived from randomized controlled trials was 0.75 (95% CI: 0.73–0.77; I^2^ = 37.8%), while that from cohort studies was 0.79 (95% CI: 0.75–0.83; I^2^ = 16.1%). These findings suggest that both study types showed a consistent inverse association between probiotic supplementation and the risk of AD, with moderate heterogeneity observed among RCTs and low heterogeneity among cohort studies.

### 3.6 Re-estimation of effect sizes and credibility ceiling analysis results

Nr, Ns, and R are 471, 73, and 32, respectively. The CCA among the included meta-analyses was calculated to be 17.6%, indicating a high degree of overlap. Due to the high degree of overlap among the included meta-analyses, removing overlapping reviews would have risked omitting key studies and introducing selection bias. Therefore, we chose to extract and synthesize all relevant original studies from the existing meta-analyses and performed a reanalysis. This approach allowed for a more comprehensive and unbiased evaluation of the evidence.

Following reanalysis of all original studies, the pooled effect estimate for the association between probiotics and the risk of AD was 0.81 (95% CI: 0.75–0.88), and 0.84 (95% CI: 0.75–0.95) for adverse events. The reanalyzed results of other subgroup comparisons are presented in Table 5. Compared with the pooled RR values from the original meta-analyses, the reanalyzed estimates showed only minor differences, suggesting a potential slight overestimation in the original results, particularly in subgroups with higher heterogeneity. Regarding adverse events, the reanalysis suggested a possible association between probiotics use and a reduced risk of adverse outcomes, whereas the original meta-analysis did not demonstrate a significant effect. This trend is consistent with the direction of the association observed for AD risk and may partially support the favorable safety profile and potential clinical value of probiotics.

**Table 5:** Results after reanalysis.

Variable	Number of study	Study design	Cases/Total	Re-evaluated RR (95% CI)	I^2^(95%CI)	Evidence credibility	RR (95% CI)
Probiotics	73	RCT, cohort	7949/42672	0.81 (0.75, 0.88)	55%(41.65%)	Highly suggestive	0.76 (0.74, 0.78)
*Lactobacillus* spp.	32	RCT	1665/6217	0.81 (0.70, 0.94)	65%(49.76%)	Weak	0.79 (0.73, 0.86)
*Bifidobacterium* spp.	3	RCT	270/868	0.89 (0.73, 1.08)	0%(0.90%)	Non-significant	0.87 (0.77, 0.99)
Mixed-strains	34	RCT, cohort	2076/7941	0.72 (0.65, 0.80)	67%(53%, 77%)	Highly suggestive	0.70 (0.65, 0.74)
Single-strain	37	RCT	6281/36306	0.82 (0.73, 0.92)	56%(37.70%)	Suggestive	0.81 (0.76, 0.86)
Prenatal	9	RCT	576/1770	0.69 (0.55, 0.88)	64%(25.82%)	Weak	0.72 (0.47, 1.09)
Postpartum	33	RCT	1960/7678	0.74 (0.64, 0.85)	64%(48.75%)	Suggestive	0.83 (0.76, 0.91)
Prenatal and postpartum	39	RCT, cohort	6823/36402	0.79 (0.73, 0.87)	61%(45.73%)	Highly suggestive	0.74 (0.71, 0.78)
Prebiotics	7	RCT	362/2256	0.77 (0.48, 1.23)	64%(17.84%)	Non-significant	0.69 (0.43, 1.13)
Synbiotics	2	RCT	148/1006	0.44 (0.11, 1.83)	–	Non-significant	0.44 (0.11, 1.79)
Adverse events	36	RCT	2187/7966	0.84 (0.75, 0.94)	54%(33, 69%)	Weak	0.95 (0.77, 1.18)

## 4 Discussion

This umbrella review represents a quantitative assessment of the association between probiotics supplementation and the risk of AD, incorporating a classification of the existing evidence. Overall, we reviewed 32 published meta-analyses, encompassing 126 comparisons. The findings of the umbrella review indicate that probiotics supplementation is associated with a lower incidence of AD, despite the presence of relatively low heterogeneity.

Currently, various probiotics, including *Bifidobacterium* spp., *Lactobacillus acidophilus*, *Lactobacillus casei*, and *Lactobacillus rhamnosus*, are widely recognized globally. However, *Lactobacillus* spp. and *Bifidobacterium* spp. are the most commonly used probiotics in clinical practice. Earlier studies have established that in healthy children, *Lactobacillus* spp. and *Bifidobacterium* spp. are the predominant species in the intestinal microbiota. Children with AD have higher quantities of *Escherichia coli* and *Staphylococcus aureus* in their intestines, while the quantities of *Bifidobacterium* spp. and *Lactobacillus* spp. are notably diminished (49, 50). This imbalance may partly account for the observed association between probiotic supplementation and a reduced risk of AD. Probiotics exert their effects through various mechanisms. Immunoglobulin A (IgA) is a crucial antimicrobial protein in intestinal mucosal defense. They prevent pathogen adhesion to the intestinal epithelium and enhance bacterial entrapment in mucus (51). *Lactobacillus* spp. and *Bifidobacterium* spp. can regulate cytokine release and modify the mucosal environment, thereby inducing IgA production and maintaining intestinal barrier integrity (52). Balancing Th1 and Th2 immune responses is recognized as one of the mechanisms of *Lactobacillus* spp. They enhance the expression of genes associated with Th1/Th2 cells, inflammatory cells, regulatory T cells, and physiological functions in the gut while reducing Th2-driven immune responses (53). *Lactobacillus* spp. enhance immune balance by upregulating IL-10 and TGF-*β* and promoting CD4 + CD25 + Foxp3 + Treg differentiation in mesenteric lymph nodes (54). *Lactobacillus* spp. also help reduce the expression of pro-inflammatory cytokines, such as IL-13, thymic stromal lymphopoietin (TSLP), and IL-5 (55, 56). On the other hand, *Bifidobacterium* spp. inhibit the growth of *Staphylococcus aureus* and *Escherichia coli* in the intestine while enhancing the production of short-chain fatty acids (SCFA) and conjugated linoleic acid (CLA) (50). This may subsequently contribute to a reduced risk of allergies. However, it is suggested that SCFAs support gut microbiota balance and are closely linked to immune cell levels (57). CLA exhibits anti-inflammatory properties and shows significant potential in alleviating AD (58). It is hypothesized that the potential benefits of probiotics are associated with the activation of Toll-like receptors (TLRs), which triggers the production of mediators such as IL-6, subsequently inducing the differentiation of naive B cells into IgA-producing cells (6). These studies provide a theoretical basis for the use of *Lactobacillus* spp. and *Bifidobacterium* spp. in the primary prevention of AD.

In this umbrella review, the findings suggest a potential association between probiotics intake and a reduced risk of AD. The strongest evidence supporting this association was classified as “highly suggestive”; however, it was derived from a meta-analysis rated as low quality by the AMSTAR 2 tool, and no association met the criteria for “convincing” evidence. Additionally, 15 (13%) comparisons were classified as having “suggestive” evidence. Amalia et al. analyzed 21 original studies, including randomized controlled trials and cohort studies, involving a total of 33,192 participants. Their results indicated that supplementation with a mixture of probiotics strains may reduce the risk of developing AD in children, regardless of high-risk status (48). However, this meta-analysis was rated as “low quality” according to the AMSTAR 2 scale. Similarly, Li Li et al. conducted a meta-analysis involving 6,892 participants and reached the same conclusion that probiotics supplementation during the prenatal and postnatal periods reduces the incidence of AD in infants and children (59). This meta-analysis was rated as “high quality” based on the AMSTAR 2 scale. While our findings may indicate a possible link between probiotics intake and reduced AD incidence, further high-quality studies are needed to strengthen the reliability of this conclusion. Compared to “convincing” evidence, the highest level of evidence we obtained is “highly suggestive.” Most of the studies included in our analysis have small sample sizes, potential small-study effects, no significant pooled effects (*p* > 10^−6^), and heterogeneity, all of which suggest that the conclusions drawn should be interpreted with caution.

We conducted a subgroup analysis based on bacterial strains. *Lactobacillus* spp. was associated with a lower risk of AD, although this conclusion is supported by “weak” evidence. Supplementation with both single-strain and multi-strain probiotics showed associations with reduced AD risk, supported by “weak” and “suggestive” evidence, respectively. Weak evidence suggests that *Lactobacillus reuteri*, alone or combined with other probiotics, appears to reduce AD incidence in pediatric patients for at least 7 years (60). This meta-analysis included 11 randomized controlled trials with a total of 2,572 participants and a maximum follow-up of 7 years, evaluating the effects of *Lactobacillus reuteri* on AD. Meta-analysis of the timeframes ≤ 2 years (RR 0.60, 95% CI 0.47–0.75; *p* < 0.00001) and 6–7 years (RR 0.62, 95% CI 0.50–0.75; p < 0.00001) both demonstrated statistically significant reductions in AD with use of *Lactobacillus rhamnosus*. These findings align with our results. The association between *Bifidobacterium* spp. and AD remains unestablished, with only three meta-analyses included in this subgroup analysis. This limited sample size is insufficient for an umbrella review, and the current evidence level remains “non-significant.”

Subgroup analyses suggest that mixed-strain probiotics may be more effective than single-strain in reducing the risk of AD. However, this finding should be interpreted with caution due to limited strength of the overall evidence and the absence of any association classified as “convincing.” The effect estimates for both mixed-and single-strain probiotics were consistent across different analytical approaches, indicating good result stability. Furthermore, after reanalyzing original studies, the evidence levels were upgraded to “highly suggestive” for mixed-strains and “suggestive” for single-strain, enhancing the reliability of these associations. A growing body of evidence also supports the superiority of mixed-strain probiotics (42, 43, 61, 62). For example, “suggestive” evidence indicates that *Lactobacillus* spp. or *Bifidobacterium* spp. alone may not significantly prevent AD in children, whereas their combination showed a significant effect (RR = 0.68, 95% CI: 0.52–0.90) (42). The enhanced efficacy of mixed-strain probiotics may result from synergistic interactions among bacterial strains, modulating the gut microbiome and immune system—an effect potentially unachievable by single-strain (63, 64). The consistent effect estimates before and after re-analysis indicate result stability, while the upgraded evidence level supports increased reliability of the association between mixed-strain probiotics and reduced AD risk. Further studies are required to validate this hypothesis.

From a mechanistic perspective, prenatal probiotics supplementation may support early fetal immune development. Pregnancy is a critical window for establishing the infant gut microbiota, and probiotics may help modulate early immune responses (65). Immune factor production may even begin before birth (66, 67). Animal studies have shown that prenatal probiotics increase IFN-*γ* levels in offspring skin (68), and human studies suggest a similar effect on fetal IFN-γ production via the feto-placental unit, though this occurs in only some infants (69). However, in our meta-analysis, the effect of prenatal supplementation appeared unstable. The effect estimate before reanalysis was 0.72 (95%CI, 0.47–1.09), and after reanalysis it was 0.69 (95%CI, 0.55–0.88). Although statistically significant, the latter was still rated as low-certainty evidence, suggesting limited robustness. In contrast, postnatal or combined prenatal-postnatal supplementation showed more consistent effects across analyses, with improved certainty levels, indicating potential value in AD prevention. Postnatal probiotics supplementation may exert the potential benefits via TGF-*β*. Animal studies have shown that cytokines in milk, such as TGF-β, can induce oral tolerance (70–72). Human milk TGF-β plays a key role in the development and maintenance of appropriate immune responses in infants and may provide protection against adverse immunological outcomes, such as AD, consistent with findings from experimental animal studies (73). Boyle et al.’s study suggests that prenatal intervention alone has no preventive effect on AD, sensitization responses, food allergies, or asthma, highlighting the importance of postnatal intervention in preventing allergic diseases (74). Moreover, the duration of prenatal-only supplementation may be too short to induce lasting effects on the infant immune system. In comparison, postnatal or combined supplementation offers a longer intervention window, potentially enhancing preventive efficacy.

In our subgroup analysis, several RRs were relatively close (e.g., 0.79 vs. 0.81). Despite these modest differences, they may still hold potential value in clinical and public health decision-making, particularly when targeting high-risk population (such as children with a family history of allergies). For example, Dale et al. conducted a study involving 28,000 mother–infant pairs to evaluate the impact of environmental exposure on mean birthweight and low birthweight. They found that vulnerable subpopulations with higher baseline risks were more adversely affected by the same environmental exposure compared to the general population (75). Similarly, Tran et al., using simulation models of different vaccine allocation strategies, showed that prioritizing high-risk groups—such as adults aged 70 and above—could substantially reduce mortality, even under constrained vaccine supply (76). Nevertheless, the clinical significance of small differences in RRs should be interpreted with caution, especially given the most evidence is rated as “weak” or “suggestive.” Clinical decision-making should incorporate not only statistical significance but also multiple factors such as effect size, certainty of evidence, disease incidence, and population characteristics.

Probiotics have been safely used for many years. Our study found that probiotics were well tolerated and were not associated with adverse events during the intervention period. Reported adverse events associated with probiotics include systemic infections, harmful metabolic activities, excessive immune stimulation in susceptible individuals, gene transfer, and gastrointestinal side effects (77). Kuang et al. demonstrated that pregnant women receiving probiotics had a significantly reduced risk of mortality and necrotizing enterocolitis, while the risks of microbiota-related symptoms, preeclampsia, and sepsis did not show statistically significant differences (78). Similarly, Cuello-Garcia et al. found no differences in adverse events between the probiotics and control groups, with most reported events being mild gastrointestinal symptoms (e.g., diarrhea, vomiting, retching, bloating), mild respiratory symptoms (e.g., cough, rhinorrhea), and mild rash (79, 80). Even in very low birth weight preterm infants, no adverse effects or complications associated with probiotics use were observed. Specifically, Lacticaseibacillus rhamnosus was not isolated from blood cultures or peritoneal fluid, and no cases of necrotizing enterocolitis (NEC) beyond stage II were reported (81). Concerns exist that probiotics could overstimulate the immune response in certain individuals, potentially triggering autoimmune phenomena or inflammation (82–84). However, this theoretical concern has not been reported in any human subjects. While horizontal gene transfer between probiotics organisms and gut microorganisms is theoretically possible (85, 86), no clinical evidence of antimicrobial resistance transfer has been documented. Probiotics have been demonstrated to provide benefits in the prevention or treatment of various pediatric diseases, such as Clostridioides difficile-associated diarrhea, infantile colic, *Helicobacter pylori* infection, necrotizing enterocolitis (NEC), and late-onset sepsis (87).

Although probiotics supplementation may offer potential benefits in the management of AD, an exclusive focus on its intake could overlook the critical role of a diverse and balanced diet in overall health maintenance. Probiotics should be appropriately integrated into a broader dietary strategy based on individualized assessment, with a primary focus on nutritional adequacy and dietary diversity. Lim et al. (88) have indicated an association between high-fiber diets and a reduced risk of AD and house dust mite allergy. Notably, moderate to high fiber intake, particularly when combined with probiotics, may further decrease the risk of developing AD. This may be related to the stabilizing effect of dietary fiber on gut microbiota diversity as well as its ability to reduce leptin levels (89, 90). Moreover, studies have shown that higher levels of short-chain fatty acids (SCFAs) in feces are significantly associated with a reduced risk of AD (91, 92). As microbial metabolites, SCFAs may exert protective effects through several mechanisms, including promoting IL-10 secretion by dendritic cells, modulating the number and function of regulatory Tregs, reducing effector T cell activity, enhancing epithelial barrier function, and inhibiting the activation of mast cells and group 2 innate lymphoid cells (93). Microbial tryptophan metabolites, such as indole-3-acetic acid, indole-3-propionic acid, and indole-3-aldehyde, can activate the aryl hydrocarbon receptor (AHR), thereby suppressing inflammatory responses and improving the epidermal skin barrier (94). It has been demonstrated that *Bifidobacterium longum* CCFM1029 metabolizes tryptophan to generate indole-3-carboxaldehyde (I3C), which activates the AHR and thereby significantly ameliorates symptoms of AD (95). In addition to microbial-derived metabolites, Flavonoids may help maintain skin barrier function by scavenging free radicals, stabilizing enzymes involved in collagen and hyaluronic acid metabolism, and enhancing skin hydration, structural integrity, and resistance to environmental irritants and allergens (96). Moreover, some studies have suggested that supplementation with dietary fats (such as gamma-linolenic acid, docosahexaenoic acid, and arachidonic acid), vitamins and pancreatic enzymes and may also exert beneficial effects on the incidence of AD in infants (97–101), although the evidence remains limited and the efficacy requires further investigation. Future research should focus on exploring synergistic dietary intervention strategies that combine probiotics with other dietary components to optimize the management of AD.

Our umbrella review possesses several strengths. First, it comprehensively synthesizes published meta-analyses on the association between probiotics supplementation and AD, representing one of the highest levels of evidence. Second, we employed a rigorous and systematic search strategy across multiple databases. Study selection and data extraction were conducted independently by two investigators. Third, we recalculated the pooled effect size for each meta-analysis using a random-effects model and assessed heterogeneity, small-study effects, and excess significance bias to facilitate a more reliable comparison of different findings. Fourth, we clarified the extent of overlap among the included studies and chose to integrate all relevant primary studies from the existing meta-analyses for re-analysis, in order to ensure the comprehensiveness and accuracy of the study’s conclusions.

However, this study has certain limitations. First, only meta-analyses with complete individual study data were included, as required by the umbrella review’s methodological framework. Consequently, relevant associations from meta-analyses with incomplete individual study data or unsynthesized studies may have been overlooked. Second, despite applying rigorous, objective criteria, inherent biases in individual studies cannot be entirely excluded. For example, the included meta-analyses did not provide a clear determination of probiotics dosage. Third, when a meta-analysis includes fewer than 10 studies, the statistical power to detect small-study effects and excess significance bias decreases, making it more challenging to identify potential sources of bias. Future large-scale randomized controlled trials with long-term follow-up are needed to generate evidence-based public health recommendations on the relationship between probiotics intake and AD.

## 5 Conclusion

Probiotics formulations are widely available and commonly used as supplements to regulate gut microbiota. This study provides a comprehensive assessment of the association between probiotics and atopic dermatitis (AD) risk. The results indicate a significant correlation between probiotics supplementation and a reduced incidence of AD. Subgroup analysis indicates that *Lactobacillus* spp., as well as both single-strain and multi-strain probiotics formulations, may contribute to risk reduction, with multi-strain preparations potentially offering greater efficacy. Furthermore, both combined prenatal and postnatal supplementation and postnatal supplementation alone were associated with decreased AD risk, underscoring the potential benefits of early-life probiotics interventions.

## Data Availability

The original contributions presented in the study are included in the article/supplementary material, further inquiries can be directed to the corresponding authors.

## References

[1] Legatzki A Rösler B von Mutius E. Microbiome diversity and asthma and allergy risk. Curr Allergy Asthma Rep. (2014) 14:466. doi: 10.1007/s11882-014-0466-0, PMID: 25149168

[2] Lee J Seto D Bielory L. Meta-analysis of clinical trials of probiotics for prevention and treatment of pediatric atopic dermatitis. J Allergy Clin Immunol. (2008) 121:116–121.e11. doi: 10.1016/j.jaci.2007.10.043, PMID: 18206506

[3] Hill C Guarner F Reid G Gibson GR Merenstein DJ Pot B . Expert consensus document. The international scientific association for probiotics and prebiotics consensus statement on the scope and appropriate use of the term probiotic. Nat Rev Gastroenterol Hepatol. (2014) 11:506–14. doi: 10.1038/nrgastro.2014.66 24912386

[4] Spergel JM. From atopic dermatitis to asthma: the atopic march. Ann Allergy Asthma Immunol. (2010) 105:99–106. doi: 10.1016/j.anai.2009.10.002, PMID: 20674819

[5] Sanders ME Guarner F Guerrant R Holt PR Quigley EMM Sartor RB . An update on the use and investigation of probiotics in health and disease. Gut. (2013) 62:787–96. doi: 10.1136/gutjnl-2012-302504, PMID: 23474420 PMC4351195

[6] Ozdemir O. Various effects of different probiotic strains in allergic disorders: an update from laboratory and clinical data. Clin Exp Immunol. (2010) 160:295–304. doi: 10.1111/j.1365-2249.2010.04109.x, PMID: 20345982 PMC2883099

[7] Novak N Yu CF Bussmann C Maintz L Peng WM Hart J . Putative association of a TLR9 promoter polymorphism with atopic eczema. Allergy. (2007) 62:766–72. doi: 10.1111/j.1398-9995.2007.01358.x, PMID: 17573724

[8] Niers L Martín R Rijkers G Sengers F Timmerman H van Uden N . The effects of selected probiotic strains on the development of eczema (the Pand a study). Allergy. (2009) 64:1349–58. doi: 10.1111/j.1398-9995.2009.02021.x, PMID: 19392993

[9] Yuejie Z. Application of probiotics in children with allergic diseases. Chin J Pract Pediatr. (2017) 32:114–7. doi: 10.19538/j.ek2017020609

[10] Thomas DW Greer FR American Academy of Pediatrics Committee on Nutrition American Academy of Pediatrics Section on Gastroenterology, Hepatology, and Nutrition. Probiotics and prebiotics in pediatrics. Pediatrics. (2010) 126:1217–31. doi: 10.1542/peds.2010-2548, PMID: 21115585

[11] Harrison SL Buckley BJR Rivera-Caravaca JM Zhang J Lip GYH. Cardiovascular risk factors, cardiovascular disease, and COVID-19: an umbrella review of systematic reviews. Eur Heart J Qual Care Clin Outcomes. (2021) 7:330–9. doi: 10.1093/ehjqcco/qcab029, PMID: 34107535 PMC8294691

[12] Veronese N Solmi M Caruso MG Giannelli G Osella AR Evangelou E . Dietary fiber and health outcomes: an umbrella review of systematic reviews and meta-analyses. Am J Clin Nutr. (2018) 107:436–44. doi: 10.1093/ajcn/nqx082, PMID: 29566200

[13] Cupp MA Cariolou M Tzoulaki I Aune D Evangelou E Berlanga-Taylor AJ. Neutrophil to lymphocyte ratio and cancer prognosis: an umbrella review of systematic reviews and meta-analyses of observational studies. BMC Med. (2020) 18:360. doi: 10.1186/s12916-020-01817-1, PMID: 33213430 PMC7678319

[14] Xu K Peng R Zou Y Jiang X Sun Q Song C. Vitamin C intake and multiple health outcomes: an umbrella review of systematic reviews and meta-analyses. Int J Food Sci Nutr. (2022) 73:588–99. doi: 10.1080/09637486.2022.2048359, PMID: 35291895

[15] Rethlefsen ML Kirtley S Waffenschmidt S Ayala AP Moher D Page MJ . PRISMA-S: an extension to the PRISMA statement for reporting literature searches in systematic reviews. Syst Rev. (2021) 10:39. doi: 10.1186/s13643-020-01542-z, PMID: 33499930 PMC7839230

[16] Shea BJ Reeves BC Wells G Thuku M Hamel C Moran J . AMSTAR 2: a critical appraisal tool for systematic reviews that include randomised or non-randomised studies of healthcare interventions, or both. BMJ. (2017) 358:j4008. doi: 10.1136/bmj.j4008, PMID: 28935701 PMC5833365

[17] Sui J Guo J Pan D Wang Y Xu Y Sun G . The efficacy of dietary intake, supplementation, and blood concentrations of carotenoids in cancer prevention: insights from an umbrella meta-analysis. Food Secur. (2024) 13:1321. doi: 10.3390/foods13091321, PMID: 38731692 PMC11083701

[18] Der Simonian R Laird N. Meta-analysis in clinical trials revisited. Contemp Clin Trials. (2015) 45:139–45. doi: 10.1016/j.cct.2015.09.002, PMID: 26343745 PMC4639420

[19] Higgins JPT Thompson SG Deeks JJ Altman DG. Measuring inconsistency in meta-analyses. BMJ. (2003) 327:557–60. doi: 10.1136/bmj.327.7414.557, PMID: 12958120 PMC192859

[20] Ioannidis JPA Patsopoulos NA Evangelou E. Uncertainty in heterogeneity estimates in meta-analyses. BMJ. (2007) 335:914–6. doi: 10.1136/bmj.39343.408449.80, PMID: 17974687 PMC2048840

[21] Riley RD Higgins JPT Deeks JJ. Interpretation of random effects meta-analyses. BMJ. (2011) 342:d549. doi: 10.1136/bmj.d549, PMID: 21310794

[22] Tsilidis KK Kasimis JC Lopez DS Ntzani EE Ioannidis JPA. Type 2 diabetes and cancer: umbrella review of meta-analyses of observational studies. BMJ. (2015) 350:g7607. doi: 10.1136/bmj.g7607, PMID: 25555821

[23] Egger M Davey Smith G Schneider M Minder C. Bias in meta-analysis detected by a simple, graphical test. BMJ. (1997) 315:629–34. doi: 10.1136/bmj.315.7109.629, PMID: 9310563 PMC2127453

[24] Belbasis L Bellou V Evangelou E Ioannidis JPA Tzoulaki I. Environmental risk factors and multiple sclerosis: an umbrella review of systematic reviews and meta-analyses. Lancet Neurol. (2015) 14:263–73. doi: 10.1016/S1474-4422(14)70267-4, PMID: 25662901

[25] Churuangsuk C Hall J Reynolds A Griffin SJ Combet E Lean MEJ. Diets for weight management in adults with type 2 diabetes: an umbrella review of published meta-analyses and systematic review of trials of diets for diabetes remission. Diabetologia. (2022) 65:14–36. doi: 10.1007/s00125-021-05577-2, PMID: 34796367 PMC8660762

[26] Solmi M Köhler CA Stubbs B Koyanagi A Bortolato B Monaco F . Environmental risk factors and nonpharmacological and nonsurgical interventions for obesity: an umbrella review of meta-analyses of cohort studies and randomized controlled trials. Eur J Clin Investig. (2018) 48:e12982. doi: 10.1111/eci.12982, PMID: 29923186

[27] Kim JY Son MJ Son CY Radua J Eisenhut M Gressier F . Environmental risk factors and biomarkers for autism spectrum disorder: an umbrella review of the evidence. Lancet Psychiatry. (2019) 6:590–600. doi: 10.1016/S2215-0366(19)30181-6, PMID: 31230684

[28] Barbui C Purgato M Abdulmalik J Acarturk C Eaton J Gastaldon C . Efficacy of psychosocial interventions for mental health outcomes in low-income and middle-income countries: an umbrella review. Lancet Psychiatry. (2020) 7:162–72. doi: 10.1016/S2215-0366(19)30511-5, PMID: 31948935

[29] Ioannidis JPA Trikalinos TA. An exploratory test for an excess of significant findings. Clin Trials. (2007) 4:245–53. doi: 10.1177/1740774507079441, PMID: 17715249

[30] Lubin JH Gail MH. On power and sample size for studying features of the relative odds of disease. Am J Epidemiol. (1990) 131:552–66. doi: 10.1093/oxfordjournals.aje.a115530, PMID: 2301364

[31] Veettil SK Wong TY Loo YS Playdon MC Lai NM Giovannucci EL . Role of diet in colorectal cancer incidence: umbrella review of meta-analyses of prospective observational studies. JAMA Netw Open. (2021) 4:e2037341. doi: 10.1001/jamanetworkopen.2020.37341, PMID: 33591366 PMC7887658

[32] Kalliala I Markozannes G Gunter MJ Paraskevaidis E Gabra H Mitra A . Obesity and gynaecological and obstetric conditions: umbrella review of the literature. BMJ. (2017) 359:j4511. doi: 10.1136/bmj.j4511, PMID: 29074629 PMC5656976

[33] Tsilidis KK Papatheodorou SI Evangelou E Ioannidis JPA. Evaluation of excess statistical significance in meta-analyses of 98 biomarker associations with cancer risk. J Natl Cancer Inst. (2012) 104:1867–78. doi: 10.1093/jnci/djs437, PMID: 23090067

[34] Chen S Su X Feng Y Li R Liao M Fan L . Ketogenic diet and multiple health outcomes: an umbrella review of meta-analysis. Nutrients. (2023) 15:4161. doi: 10.3390/nu15194161, PMID: 37836444 PMC10574428

[35] Pieper D Antoine SL Mathes T Neugebauer EAM Eikermann M. Systematic review finds overlapping reviews were not mentioned in every other overview. J Clin Epidemiol. (2014) 67:368–75. doi: 10.1016/j.jclinepi.2013.11.007, PMID: 24581293

[36] Solmi M De Toffol M Kim JY Choi MJ Stubbs B Thompson T . Balancing risks and benefits of cannabis use: umbrella review of meta-analyses of randomised controlled trials and observational studies. BMJ. (2023) 382:e072348. doi: 10.1136/bmj-2022-072348, PMID: 37648266 PMC10466434

[37] Higgins JPT Thomas J Chandler J Cumpston M Li T Page MJ Cochrane handbook for systematic reviews of interventions version 6.4 (updated August 2023). Cochrane; (2023). Available online at: https://training.cochrane.org/handbook

[38] Cooper H Koenka AC. The overview of reviews: unique challenges and opportunities when research syntheses are the principal elements of new integrative scholarship. Am Psychol. (2012) 67:446–62. doi: 10.1037/a0027119, PMID: 22352742

[39] Huang Y Chen Z Chen B Li J Yuan X Li J . Dietary sugar consumption and health: umbrella review. BMJ. (2023) 381:e071609. doi: 10.1136/bmj-2022-071609, PMID: 37019448 PMC10074550

[40] Xie Y Xu J Zhou D Guo M Zhang M Gao Y . Micronutrient perspective on COVID-19: umbrella review and reanalysis of meta-analyses. Crit Rev Food Sci Nutr. (2024) 64:6783–801. doi: 10.1080/10408398.2023.2174948, PMID: 36794398

[41] Jaramillo-Rodríguez OD González-Correa CH. Probióticos en prevención primaria de la dermatitis atópica en infantes con riesgo de padecerla: Metaanálisis. Biosalud. (2013) 12:18–28. Available at: http://www.scielo.org.co/scielo.php?script=sci_arttext&pid=S1657-95502013000100003&lng=en

[42] Yin DG He Z Duan XY Fan FX Liao XB Wang QC. Effect of probiotic supplementation during pregnancy and infancy in preventing atopic dermatitis in children: a meta analysis. Zhongguo Dang Dai Er Ke Za Zhi. (2019) 21:82–8. doi: 10.7499/j.issn.1008-8830.2019.01.015 PMID: 30675869 PMC7390177

[43] Zhu DL Yang WX Yang HM. Meta analysis of lactic acid bacteria as probiotics for the primary prevention of infantile eczema. Zhongguo Dang Dai Er Ke Za Zhi. (2010) 12:734–9. 20849726

[44] Tang LJ Chen J Shen Y. Meta-analysis of probiotics preventing allergic diseases in infants. Zhonghua Er Ke Za Zhi. (2012) 50:504–9. 22932010

[45] Cao L Wang L Yang L Tao S Xia R Fan W. Long-term effect of early-life supplementation with probiotics on preventing atopic dermatitis: a meta-analysis. J Dermatolog Treat. (2015) 26:537–40. doi: 10.3109/09546634.2015.1027168, PMID: 25942569

[46] Wang F Wu F Chen H Tang B. The effect of probiotics in the prevention of atopic dermatitis in children: a systematic review and meta-analysis. Transl Pediatr. (2023) 12:731–48. doi: 10.21037/tp-23-200, PMID: 37181018 PMC10167384

[47] Liang Hong Lin Liang HL Liu Jing Liu LJ Wu Bin Wu WB. Preventative effect of probiotics for infantile atopic dermatitis: a systematic review. (2018). Available online at: https://www.cabidigitallibrary.org/doi/full/10.5555/20183361278

[48] Amalia N Orchard D Francis KL King E. Systematic review and meta-analysis on the use of probiotic supplementation in pregnant mother, breastfeeding mother and infant for the prevention of atopic dermatitis in children. Australas J Dermatol. (2020) 61:e158–73. doi: 10.1111/ajd.13186, PMID: 31721162

[49] West CE Jenmalm MC Prescott SL. The gut microbiota and its role in the development of allergic disease: a wider perspective. Clin Exp Allergy. (2015) 45:43–53. doi: 10.1111/cea.12332, PMID: 24773202

[50] Fang Z Li L Zhang H Zhao J Lu W Chen W. Gut microbiota, probiotics, and their interactions in prevention and treatment of atopic dermatitis: a review. Front Immunol. (2021) 12:720393. doi: 10.3389/fimmu.2021.720393, PMID: 34335634 PMC8317022

[51] Chairatana P Nolan EM. Defensins, lectins, mucins, and secretory immunoglobulin a: microbe-binding biomolecules that contribute to mucosal immunity in the human gut. Crit Rev Biochem Mol Biol. (2017) 52:45–56. doi: 10.1080/10409238.2016.1243654, PMID: 27841019 PMC5233583

[52] Hardy H Harris J Lyon E Beal J Foey AD. Probiotics, prebiotics and immunomodulation of gut mucosal defences: homeostasis and immunopathology. Nutrients. (2013) 5:1869–912. doi: 10.3390/nu5061869, PMID: 23760057 PMC3725482

[53] Fu G Zhao K Chen H Wang Y Nie L Wei H . Effect of 3 lactobacilli on immunoregulation and intestinal microbiota in a β-lactoglobulin-induced allergic mouse model. J Dairy Sci. (2019) 102:1943–58. doi: 10.3168/jds.2018-15683, PMID: 30660420

[54] Kwon MS Lim SK Jang JY Lee J Park HK Kim N . *Lactobacillus sakei* WIKIM30 ameliorates atopic dermatitis-like skin lesions by inducing regulatory T cells and altering gut microbiota structure in mice. Front Immunol. (2018) 9:1905. doi: 10.3389/fimmu.2018.01905, PMID: 30154801 PMC6102352

[55] Jeong DY Ryu MS Yang HJ Jeong SY Zhang T Yang HJ . *Pediococcus acidilactici* intake decreases the clinical severity of atopic dermatitis along with increasing mucin production and improving the gut microbiome in nc/nga mice. Biomed Pharmacother. (2020) 129:110488. doi: 10.1016/j.biopha.2020.110488, PMID: 32768968

[56] Ménard S Laharie D Asensio C Vidal-Martinez T Candalh C Rullier A . Bifidobacterium breve and *streptococcus thermophilus* secretion products enhance T helper 1 immune response and intestinal barrier in mice. Exp Biol Med (Maywood). (2005) 230:749–56. doi: 10.1177/153537020523001008, PMID: 16246902

[57] Smith PM Howitt MR Panikov N Michaud M Gallini CA Bohlooly-Y M . The microbial metabolites, short-chain fatty acids, regulate colonic treg cell homeostasis. Science. (2013) 341:569–73. doi: 10.1126/science.1241165, PMID: 23828891 PMC3807819

[58] Tang L Li XL Deng ZX Xiao Y Cheng YH Li J . Conjugated linoleic acid attenuates 2, 4-dinitrofluorobenzene-induced atopic dermatitis in mice through dual inhibition of COX-2/5-LOX and TLR4/NF-κB signaling. J Nutr Biochem. (2020) 81:108379. doi: 10.1016/j.jnutbio.2020.108379, PMID: 32330842

[59] Li L Han Z Niu X Zhang G Jia Y Zhang S . Probiotic supplementation for prevention of atopic dermatitis in infants and children: a systematic review and meta-analysis. Am J Clin Dermatol. (2019) 20:367–77. doi: 10.1007/s40257-018-0404-3, PMID: 30465329

[60] Voigt J Lele M. *Lactobacillus rhamnosus* used in the perinatal period for the prevention of atopic dermatitis in infants: a systematic review and meta-analysis of randomized trials. Am J Clin Dermatol. (2022) 23:801–11. doi: 10.1007/s40257-022-00723-x, PMID: 36161401 PMC9576646

[61] Zuccotti G Meneghin F Aceti A Barone G Callegari ML Di Mauro A . Probiotics for prevention of atopic diseases in infants: systematic review and meta-analysis. Allergy. (2015) 70:1356–71. doi: 10.1111/all.12700, PMID: 26198702

[62] Dang D Zhou W Lun ZJ Mu X Wang DX Wu H. Meta-analysis of probiotics and/or prebiotics for the prevention of eczema. J Int Med Res. (2013) 41:1426–36. doi: 10.1177/0300060513493692, PMID: 23908398

[63] Chang YS Trivedi MK Jha A Lin YF Dimaano L García-Romero MT. Synbiotics for prevention and treatment of atopic dermatitis: a meta-analysis of randomized clinical trials. JAMA Pediatr. (2016) 170:236–42. doi: 10.1001/jamapediatrics.2015.3943, PMID: 26810481

[64] Jiang W Ni B Liu Z Liu X Xie W Wu IXY . The role of probiotics in the prevention and treatment of atopic dermatitis in children: an updated systematic review and meta-analysis of randomized controlled trials. Paediatr Drugs. (2020) 22:535–49. doi: 10.1007/s40272-020-00410-6, PMID: 32748341

[65] Sun M Luo J Liu H Xi Y Lin Q. Can mixed strains of lactobacillus and bifidobacterium reduce eczema in infants under three years of age? A meta-analysis. Nutrients. (2021) 13:1461. doi: 10.3390/nu13051461, PMID: 33923096 PMC8145948

[66] Jones CA Holloway JA Warner JO. Does atopic disease start in foetal life? Allergy. (2000) 55:2–10. doi: 10.1034/j.1398-9995.2000.00109.x, PMID: 10696851

[67] Landreth KS. Critical windows in development of the rodent immune system. Hum Exp Toxicol. (2002) 21:493–8. doi: 10.1191/0960327102ht287oa. 12458906

[68] Tanaka A Jung K Benyacoub J Prioult G Okamoto N Ohmori K . Oral supplementation with *lactobacillus rhamnosus* CGMCC 1.3724 prevents development of atopic dermatitis in NC/Nga Tnd mice possibly by modulating local production of IFN-gamma. Exp Dermatol. (2009) 18:1022–7. doi: 10.1111/j.1600-0625.2009.00895.x, PMID: 19555432

[69] Prescott SL Wickens K Westcott L Jung W Currie H Black PN . Supplementation with *lactobacillus rhamnosus* or *bifidobacterium lactis* probiotics in pregnancy increases cord blood interferon-gamma and breast milk transforming growth factor-beta and immunoglobin a detection. Clin Exp Allergy. (2008) 38:1606–14. doi: 10.1111/j.1365-2222.2008.03061.x, PMID: 18631345

[70] Penttila IA van Spriel AB Zhang MF Xian CJ Steeb CB Cummins AG . Transforming growth factor-beta levels in maternal milk and expression in postnatal rat duodenum and ileum. Pediatr Res. (1998) 44:524–31. doi: 10.1203/00006450-199810000-00010, PMID: 9773841

[71] Penttila IA Flesch IEA McCue AL Powell BC Zhou FH Read LC . Maternal milk regulation of cell infiltration and interleukin 18 in the intestine of suckling rat pups. Gut. (2003) 52:1579–86. doi: 10.1136/gut.52.11.1579, PMID: 14570726 PMC1773864

[72] Penttila I. Effects of transforming growth factor-beta and formula feeding on systemic immune responses to dietary beta-lactoglobulin in allergy-prone rats. Pediatr Res. (2006) 59:650–5. doi: 10.1203/01.pdr.0000203149.75465.74, PMID: 16627876

[73] Oddy WH Rosales F. A systematic review of the importance of milk TGF-beta on immunological outcomes in the infant and young child. Pediatr Allergy Immunol. (2010) 21:47–59. doi: 10.1111/j.1399-3038.2009.00913.x 19594862

[74] Boyle RJ Ismail IH Kivivuori S Licciardi PV Robins-Browne RM Mah L-J . Lactobacillus GG treatment during pregnancy for the prevention of eczema: a randomized controlled trial. Allergy. (2011) 66:509–16. doi: 10.1111/j.1398-9995.2010.02507.x, PMID: 21121927

[75] Peacock JL Coto SD Rees JR Sauzet O Jensen ET Fichorova R . Do small effects matter more in vulnerable populations? An investigation using environmental influences on child health outcomes (ECHO) cohorts. BMC Public Health. (2024) 24:2655. doi: 10.1186/s12889-024-20075-x, PMID: 39342237 PMC11438038

[76] Tran TNA Wikle NB Albert E Inam H Strong E Brinda K . Optimal SARS-CoV-2 vaccine allocation using real-time attack-rate estimates in rhode Island and Massachusetts. BMC Med. (2021) 19:162. doi: 10.1186/s12916-021-02038-w, PMID: 34253200 PMC8275456

[77] Doron S Snydman DR. Risk and safety of probiotics. Clin Infect Dis. (2015) 60:S129–34. doi: 10.1093/cid/civ085, PMID: 25922398 PMC4490230

[78] Kuang L Jiang Y. Effect of probiotic supplementation in pregnant women: a meta-analysis of randomised controlled trials. Br J Nutr. (2020) 123:870–80. doi: 10.1017/S0007114519003374, PMID: 31856928

[79] Cuello-Garcia C Fiocchi A Pawankar R Yepes-Nuñez JJ Morgano GP Zhang Y . Prebiotics for the prevention of allergies: a systematic review and meta-analysis of randomized controlled trials. Clin Exp Allergy. (2017) 47:1468–77. doi: 10.1111/cea.13042, PMID: 29035013

[80] Cuello-Garcia CA Brożek JL Fiocchi A Pawankar R Yepes-Nuñez JJ Terracciano L . Probiotics for the prevention of allergy: a systematic review and meta-analysis of randomized controlled trials. J Allergy Clin Immunol. (2015) 136:952–61. doi: 10.1016/j.jaci.2015.04.031, PMID: 26044853

[81] Uberos J Garcia-Cuesta A Carrasco-Solis M Ruiz-López A Fernandez-Marı NE Campos-Martinez A. Lacticaseibacillus rhamnosus and breastmilk are associated with a decreased risk of atopic dermatitis in very low birth weight premature infants. Benef Microbes. (2023) 14:433–43. doi: 10.1163/18762891-20220144, PMID: 38656097

[82] Veckman V Miettinen M Pirhonen J Sirén J Matikainen S Julkunen I. Streptococcus pyogenes and *lactobacillus rhamnosus* differentially induce maturation and production of Th1-type cytokines and chemokines in human monocyte-derived dendritic cells. J Leukoc Biol. (2004) 75:764–71. doi: 10.1189/jlb.1003461, PMID: 14966192

[83] Drakes M Blanchard T Czinn S. Bacterial probiotic modulation of dendritic cells. Infect Immun. (2004) 72:3299–309. doi: 10.1128/IAI.72.6.3299-3309.2004, PMID: 15155633 PMC415669

[84] Braat H de Jong EC van den Brande JMH Kapsenberg ML Peppelenbosch MP van Tol EAF . Dichotomy between lactobacillus rhamnosus and klebsiella pneumoniae on dendritic cell phenotype and function. J Mol Med (Berl). (2004) 82:197–205. doi: 10.1007/s00109-003-0509-9, PMID: 14673529

[85] Dessart SR Steenson LR. High frequency intergeneric and intrageneric conjugal transfer of drug resistance plasmids in *Leuconostoc mesenteroides* ssp. cremoris. J Dairy Sci. (1991) 74:2912–9.

[86] Morelli L Sarra PG Bottazzi V. In vivo transfer of pAM beta 1 from *lactobacillus reuteri* to *enterococcus faecalis*. J Appl Bacteriol. (1988) 65:371–5. doi: 10.1111/j.1365-2672.1988.tb01905.x, PMID: 2976754

[87] Depoorter L Vandenplas Y. Probiotics in pediatrics. A review and practical guide. Nutrients. (2021) 13:2176. doi: 10.3390/nu13072176, PMID: 34202742 PMC8308463

[88] Lim JJ Reginald K Say YH Liu MH Chew FT. Frequent intake of high fiber and probiotic diets lowers risks associated with atopic dermatitis and house dust mite allergy: a cross-sequential study of young Chinese adults from Singapore and Malaysia. Eur J Nutr. (2024) 64:38. doi: 10.1007/s00394-024-03524-6, PMID: 39614888 PMC11608386

[89] Swann OG Breslin M Kilpatrick M O’Sullivan TA Mori TA Beilin LJ . Dietary fibre intake and its association with inflammatory markers in adolescents. Br J Nutr. (2021) 125:329–36. doi: 10.1017/S0007114520001609, PMID: 32378492

[90] Tap J Furet JP Bensaada M Philippe C Roth H Rabot S . Gut microbiota richness promotes its stability upon increased dietary fibre intake in healthy adults. Environ Microbiol. (2015) 17:4954–64. doi: 10.1111/1462-2920.13006, PMID: 26235304

[91] Song H Yoo Y Hwang J Na Y-C Kim HS. *Faecalibacterium prausnitzii* subspecies-level dysbiosis in the human gut microbiome underlying atopic dermatitis. J Allergy Clin Immunol. (2016) 137:852–60. doi: 10.1016/j.jaci.2015.08.021 26431583

[92] Roduit C Frei R Ferstl R Loeliger S Westermann P Rhyner C . High levels of butyrate and propionate in early life are associated with protection against atopy. Allergy. (2019) 74:799–809. doi: 10.1111/all.13660 30390309

[93] Forde B Yao L Shaha R Murphy S Lunjani N O’Mahony L. Immunomodulation by foods and microbes: unravelling the molecular tango. Allergy. (2022) 77:3513–26. doi: 10.1111/all.15455 35892227 PMC10087875

[94] Powell DN Swimm A Sonowal R Bretin A Gewirtz AT Jones RM . Indoles from the commensal microbiota act via the AHR and IL-10 to tune the cellular composition of the colonic epithelium during aging. Proc Natl Acad Sci USA. (2020) 117:21519–26. doi: 10.1073/pnas.2003004117, PMID: 32817517 PMC7474656

[95] Fang Z Pan T Li L Wang H Zhu J Zhang H . *Bifidobacterium longum* mediated tryptophan metabolism to improve atopic dermatitis via the gut-skin axis. Gut Microbes. (2022) 14:2044723. doi: 10.1080/19490976.2022.2044723 PMC8903757 35239463

[96] Zawawi NA Ahmad H Madatheri R Fadilah NIM Maarof M Fauzi MB. Flavonoids as natural anti-inflammatory agents in the atopic dermatitis treatment. Pharmaceutics. (2025) 17:261. doi: 10.3390/pharmaceutics17020261, PMID: 40006628 PMC11859288

[97] Lim JJ Liu MH Chew FT. Dietary interventions in atopic dermatitis: a comprehensive scoping review and analysis. Int Arch Allergy Immunol. (2024) 185:545–89. doi: 10.1159/000535903, PMID: 38442688 PMC11151999

[98] Dotterud CK Storrø O Simpson MR Johnsen R Øien T. The impact of pre-and postnatal exposures on allergy related diseases in childhood: a controlled multicentre intervention study in primary health care. BMC Public Health. (2013) 13:123. doi: 10.1186/1471-2458-13-123, PMID: 23394141 PMC3582458

[99] Birch EE Khoury JC Berseth CL Castañeda YS Couch JM Bean J . The impact of early nutrition on incidence of allergic manifestations and common respiratory illnesses in children. J Pediatr. (2010) 156:902–906.e1. doi: 10.1016/j.jpeds.2010.01.002, PMID: 20227721

[100] van Gool CJAW Thijs C Henquet CJM van Houwelingen AC Dagnelie PC Schrander J . Gamma-linolenic acid supplementation for prophylaxis of atopic dermatitis--a randomized controlled trial in infants at high familial risk. Am J Clin Nutr. (2003) 77:943–51. doi: 10.1093/ajcn/77.4.943 12663296

[101] Hederos CA Berg A. Epogam evening primrose oil treatment in atopic dermatitis and asthma. Arch Dis Child. (1996) 75:494–7. doi: 10.1136/adc.75.6.494, PMID: 9014601 PMC1511801

[102] Wang S Yin P Yu L Tian F Chen W Zhai Q. Effects of early diet on the prevalence of allergic disease in children: a systematic review and meta-analysis. Adv Nutr. (2024) 15:100128. doi: 10.1016/j.advnut.2023.10.001, PMID: 37827490 PMC10831899

[103] Husein-ElAhmed H Steinhoff M. Meta-analysis on preventive and therapeutic effects of probiotic supplementation in infant atopic dermatitis. J Dtsch Dermatol Ges. (2023) 21:833–43. doi: 10.1111/ddg.15120, PMID: 37345893

[104] Sun S Chang G Zhang L. The prevention effect of probiotics against eczema in children: an update systematic review and meta-analysis. J Dermatolog Treat. (2022) 33:1844–54. doi: 10.1080/09546634.2021.1925077, PMID: 34006167

[105] Pan H Su J. Association of probiotics with atopic dermatitis among infant: a meta-analysis of randomized controlled trials. Oxidative Med Cell Longev. (2022) 2022:5080190. doi: 10.1155/2022/5080190, PMID: 35651728 PMC9150986

[106] Chen L Ni Y Wu X Chen G. Probiotics for the prevention of atopic dermatitis in infants from different geographic regions: a systematic review and meta-analysis. J Dermatolog Treat. (2022) 33:2931–9. doi: 10.1080/09546634.2022.2091101, PMID: 35708329

[107] Szajewska H Horvath A. *Lactobacillus rhamnosus* GG in the primary prevention of eczema in children: a systematic review and meta-analysis. Nutrients. (2018) 10:1319. doi: 10.3390/nu10091319, PMID: 30231505 PMC6163317

[108] Panduru M Panduru NM Sălăvăstru CM Tiplica GS. Probiotics and primary prevention of atopic dermatitis: a meta-analysis of randomized controlled studies. J Eur Acad Dermatol Venereol. (2015) 29:232–42. doi: 10.1111/jdv.12496, PMID: 24698503

[109] Mansfield JA Bergin SW Cooper JR Olsen CH. Comparative probiotic strain efficacy in the prevention of eczema in infants and children: a systematic review and meta-analysis. Mil Med. (2014) 179:580–92. doi: 10.7205/MILMED-D-13-00546, PMID: 24902123

[110] Pelucchi C Chatenoud L Turati F Galeone C Moja L Bach JF . Probiotics supplementation during pregnancy or infancy for the prevention of atopic dermatitis: a meta-analysis. Epidemiology. (2012) 23:402–14. doi: 10.1097/EDE.0b013e31824d5da2, PMID: 22441545

[111] Wang Y Zeng G Zhu C Huang LJ Liu K Yu L. Preventative effects of probiotics for infantile eczema and atopic eczema: a systematic review. Chin J Evid Based Med. (2012) 12:1372–8. doi: 10.7507/1672-2531.20120214

[112] Doege K Grajecki D Zyriax BC Detinkina E Zu Eulenburg C Buhling KJ. Impact of maternal supplementation with probiotics during pregnancy on atopic eczema in childhood--a meta-analysis. Br J Nutr. (2012) 107:1–6. doi: 10.1017/S0007114511003400, PMID: 21787448

[113] Da O Jkh S. Prebiotics in infants for prevention of allergic disease and food hypersensitivity. Cochrane Database Systematic Review. (2009). 10.1002/14651858.CD006474.pub2 17943911

[114] Osborn DA Sinn JK. Probiotics in infants for prevention of allergic disease and food hypersensitivity. Cochrane Database Syst Rev. (2007) 4:CD006475. doi: 10.1002/14651858.CD006475.pub2 17943912

